# The Role of Chemokines in the Pathogenesis of HTLV-1

**DOI:** 10.3389/fmicb.2020.00421

**Published:** 2020-03-13

**Authors:** Razieh Zargari, Maryam Mahdifar, Asadollah Mohammadi, Zohreh Vahidi, Gholamhossein Hassanshahi, Houshang Rafatpanah

**Affiliations:** ^1^Immunology Research Center, Inflammation and Inflammatory Diseases Division, Mashhad University of Medical Sciences, Mashhad, Iran; ^2^Cellular and Molecular Research Center, Research Institute for Health Development, Kurdistan University of Medical Sciences, Sanandaj, Iran; ^3^Molecular Medicine Research Center, Rafsanjan University of Medical Sciences, Rafsanjan, Iran

**Keywords:** chemokine, HTLV-1, ATL, HAM/TSP, inflammatory response

## Abstract

Human T cell leukemia virus type 1 (HTLV-1) is a human retrovirus that is associated with two main diseases: HTLV-associated myelopathy/tropical spastic paraparesis (HAM/TSP) and adult T cell leukemia/lymphoma (ATL). Chemokines are highly specialized groups of cytokines that play important roles in organizing, trafficking, homing, and in the migration of immune cells to the bone marrow, lymphoid organs and sites of infection and inflammation. Aberrant expression or function of chemokines, or their receptors, has been linked to the protection against or susceptibility to specific infectious diseases, as well as increased the risk of autoimmune diseases and malignancy. Chemokines and their receptors participate in pathogenesis of HTLV-1 associated diseases from inflammation in the central nervous system (CNS) which occurs in cases of HAM/TSP to T cell immortalization and tissue infiltration observed in ATL patients. Chemokines represent viable effective prognostic biomarkers for HTLV-1-associated diseases which provide the early identification of high-risk, treatment possibilities and high-yielding clinical trials. This review focuses on the emerging roles of these molecules in the outcome of HTLV-1-associated diseases.

## Introduction

Human T cell leukemia virus type 1 (HTLV-1) belongs to the Retroviridae family, the orthoretrovirinae subfamily and to the deltaretrovirus genus (Poiesz et al., 1980). HTLV-1 has been recognized as a causative agent of two important diseases, HTLV-1 associated myelopathy/tropical spastic paraparesis (HAM/TSP) and adult T cell leukemia/lymphoma (ATL) (Yoshida et al., 1982; Gessain et al., 1985; Osame et al., 1986). The virus not only induces HAM/TSP and ATL in a small proportion of HTLV-1 carriers, but it is also associated with several diseases such as HTLV-1 associated arthropathy (HAAP), exocrinopathy, HTLV-1 uveitis (HU), cutaneous T cell lymphoma (CTCL), dermatitis, Graves’ disease, lymphadenitis, polymyositis, and chronic respiratory diseases (Kimura et al., 1986; Green et al., 1989; Morgan et al., 1989; Nishioka et al., 1989; LaGrenade et al., 1990; Hall et al., 1991; Mochizuki et al., 1992; Ohshima et al., 1992; Yamaguchi et al., 1994).

The exact mechanism of the development and pathogenesis of HAM/TSP and ATL among less than 3–5% of HTLV-1 carriers has not fully understood. However, it has been reported that genetic background such as human leukocyte antigen (HLA) and HTLV-1 genomic variation are associated with the risk of HAM/TSP among HTLV-1 carriers (Mahieux et al., 1995; Jeffery et al., 1999; Furukawa et al., 2000; Sabouri et al., 2005; Rafatpanah et al., 2007). The major criteria that differ HAM/TSP patients from HTLV-1-infected ACs are high titers of anti-HTLV-1 antibody, increased proviral loads, higher frequency of HTLV-1 specific cytotoxic T lymphocytes (CTLs), and elevation in activated lymphocytes (Kira et al., 1992; Elovaara et al., 1993; Nagai et al., 1998). Evidence also suggests viral and environmental factors, the efficiency of immune response and the genetic background of the HTLV-1 infected individual affect the outcome of HTLV-1 infection.

In HAM/TSP patients, high CTL responses may lead to the production of inflammatory cytokines and bystander damage. Based on this criterion, HTLV-1 specific CD8^+^ clones in HAM/TSP patients produce a wide variety of members of cyto/chemokine family, such as Interferon-gamma (IFN-γ), Tumor necrosis factor-alpha (TNF-α), Interleukin (IL)-2, Macrophage inflammatory protein 1-alpha (MIP-1α)/CCL3, beta (MIP-1β)/CCL4, and Matrix metalloproteinase-9 (MMP-9), which promote the inflammatory response (Elovaara et al., 1993). HTLV-1 infected cells have the potential to produce IFN-γ and migration toward tissues, including central nervous system (CNS), which may induce a harmful non-specific immune response within the CNS (Elovaara et al., 1993). Furthermore, Tax-inducible production of TNF-α, IL-1β, and IL-6 by macrophages and microglia may enhance the proinflammatory response in the CNS (Giraudon et al., 1995; Ahuja et al., 2007).

The immune response and the number of infected cells in the cerebrospinal fluid (CSF) are important factors for the induction of chronic inflammation observed in the CNS of HAM/TSP patients (Bangham et al., 2015; Kubota, 2017). Plasma level of CCL24 that is related to type 2 immune response has been reported to be reduced in HAM/TSP. The low CCL24 plasma levels support the theory of enhanced type 1 immune response in HAM/TSP patients (Guerreiro et al., 2006). It seems that the number of chemokines and their receptors in the peripheral blood and in the site of inflammation act as a risk factor for the development of HAM/TSP. Previous studies have revealed that chemokines and their receptors play a critical role in HTLV-1 infection and migration of HTLV-1 infected cells into the CNS through the blood-brain barrier (BBB); playing an essential role in the onset of HAM/TSP (Ando et al., 2013). It was also shown that cerebral endothelial cells are susceptible to HTLV-1 infection, leading to HTLV-1 passage through the BBB and subsequent BBB breakdown (Afonso et al., 2007, 2008).

ATL is a peripheral T-cell malignancy with poor prognosis (Katsuya et al., 2015). In contrast to HAM/TSP, ATL patients exhibit immunosuppressive conditions (Uchiyama et al., 1977; Bunn et al., 1983; Shimoyama and Group, 1991). The immune system in ATL patients is directly suppressed by cytokines such as IL-10 and transforming growth factor-beta (TGF-B), which might be attributed to IL-10 production by Tax and HTLV-1 basic leucine zipper factor (HBZ) promotion (Mori et al., 1996; Inagaki et al., 2006; Matsuoka and Yasunaga, 2013).

Studies have shown that ATL cells obtained from peripheral blood and skin tumors of ATL patients are CD4^+^CD25^+^ FOxp3^low^ and express cytotoxic T lymphocyte-associated antigen (CTLA)-4 with no regulatory activity (Shimauchi et al., 2008; Satou et al., 2012). Therefore, the pattern of cytokine production provides evidence for the pathogenesis of HTLV-1 associated diseases. The other factor, which is involved in the migration and tissue microenvironmental localization of lymphocytes, is adhesion molecules that are expressed on the surface of certain cells (El-Sabban et al., 2002; Banerjee et al., 2007). Furthermore, chemokines and their associated receptors are also involved in the pathogenesis of ATL as it infiltrates ATL cells into the lymph nodes, spleen, liver, skin and GIT (Hasegawa et al., 2000; Bangham et al., 2015; Kubota, 2017). Here, we discuss the role of chemokines and their receptors in the development and pathogenesis of HAM/TSP, ATL and other HTLV-1 related diseases.

## Chemokines and Their Receptors

Chemokines or chemotactic cytokines are a group of small molecules (weighing ∼8–14 kDa) which regulate migration and trafficking of various types of immune cells through interactions with a subset of cell surface seven-transmembrane G protein-coupled receptors (GPCRs) (Zlotnik and Yoshie, 2000). To date, about 50 chemokines and 20 chemokine receptors have been identified in humans (Blanchet et al., 2012). Based on a new classification, chemokine ligands and receptors are named as ‘L’ and ‘R,’ respectively (Zlotnik and Yoshie, 2000). Chemokines play pivotal roles in the development, homeostasis, and functions of the immune system (Zlotnik and Yoshie, 2000). They mainly act to traffic monocytes, lymphocytes, neutrophils, and eosinophils to the site of injury, infection, and inflammation, and thus play an important role in host defense mechanisms (Zlotnik and Yoshie, 2000). Chemokines are involved in the pathobiology of chronic inflammations, autoimmune diseases, tumorigenesis, and tumor metastasis. In fact, a disease often occurs when the physiological role of chemokines is disrupted (Raman et al., 2011).

In addition to their role in the immune system, chemokines also have a wide range of effects on many different cell types including various types of the CNS or endothelial cells, where they result in either angiogenesis or angiostasis (Strieter et al., 1995; Ma et al., 1998). They are involved in cell migration during embryogenesis and also play important roles in wound healing, angiogenesis/angiostasis and tumor development and metastasis (McGrath et al., 1999; Mashino et al., 2002; Li et al., 2004; Yang and Richmond, 2004; Burns et al., 2006; Ishida et al., 2008). The role played by chemokine/receptor axes in tumor pathophysiology is complex as some chemokines enhance tumor development and metastasis, while others associate with an effective anti-tumor immune response (Singh et al., 2007).

On the basis of the arrangement of the two N-terminal cysteine residues in their biochemical structure, chemokines are further divided into four major subfamilies, CXC, CX3C, CC, and C, depending on whether the first two cysteine residues have either one or more amino acids between them (CXC, CX3C) or are adjacent (CC) (Miller and Mayo, 2017). CXC chemokines have the ability to activate and attract neutrophils and T lymphocytes, while CC chemokines are effective on multiple leukocyte subtypes including monocytes, eosinophils, T lymphocytes, basophils, dendritic cells (DCs), natural killer (NK) cells, and neutrophils. CX3C (Fraktaline) induces the migration of T cells, NK cells, and monocytes (Luster, 1998; Lee et al., 2018). Since the receptors for CC and CXC chemokines determine the viral tropism and mediate the efficient entry of human immunodeficiency virus (HIV) into host cells, CC and CXC chemokines could suppress HIV infection (Owais and Arya, 1999). CC chemokines mostly inhibit the entry and replication of macrophage-tropic strains; nonetheless, CXC chemokines mostly inhibit the entry of T-tropic strains (Owais and Arya, 1999).

According to their function and pattern of expression, chemokines have also been classified into homeostatic and inflammatory groups or both. The homeostatic chemokines are constitutively expressed and important in many physiological processes and are involved in normal circulation and trafficking of leukocytes in lymph nodes and thymus, while the production of inflammatory group is induced by infection or other proinflammatory stimuli resulting in recruitment of leukocytes to the insulted regions, in particular, due to infection (Cyster, 1999; Sharma, 2010).

Chemokine receptors are subdivided into CCR, CXCR, CX3CR, and CR based on the ligand to which they bind. The interesting fact about the chemokine receptors is the great level of redundancy observed in their effects on targets in which most of them can bind to several chemokines and most chemokines utilize more than one receptor (Zlotnik and Yoshie, 2000). During viral infection, the expression of CC chemokines is more prevalent than CXC chemokines (Melchjorsen et al., 2003; Dahm et al., 2016). CCL3 and CCL5 appear to be mostly related to viral infections (Rösler et al., 1998; Bonville et al., 1999; Haeberle et al., 2001; Domachowske et al., 2002). Among CXCL chemokines which are produced during viral infection, CXCL10 expression is observed in many viral infections (Dawson et al., 2000; Haeberle et al., 2001; Liu et al., 2001; Peterson et al., 2001). CCL2 and CXCL10 are expressed at large measures during human viral meningitis and influenza infections, while in the case of hepatitis C virus (HCV), CCL5 and CXCL9 are more expressed (Lahrtz et al., 1997; Dawson et al., 2000; Haeberle et al., 2001; Apolinario et al., 2002).

## Chemokines and HTLV-1 Viral Transcriptional Regulators

HTLV-1 Tax and HBZ viral products are associated with oncogenic transformation and ATL pathogenesis (Pozzatti et al., 1990; Tanaka et al., 1990; Satou et al., 2006). Tax is a key regulatory protein encoded by the pX region of the HTLV-1 genome, while HBZ is encoded by the antisense strand of the virus genome (Nagashima et al., 1986; Larocca et al., 1989; Cavanagh et al., 2006). Tax and HBZ regulate transcription of viral proteins, host factors and cellular signaling pathways, resulting in development of HTLV-1 associated diseases (Currer et al., 2012; Zhao, 2016). The HTLV-1 Tax protein has been known as a potent activator of a variety of transcription factors including NF-kB, CREB, SRF, and AP-1 (Fujii et al., 1992, 1995, 2000; Bex et al., 1998; Jeang, 2001). It was shown that the transcription factors NF-kB and AP-1 are involved in Tax-mediated transactivation of IL-8 (Mori et al., 1998). Extracellular Tax has the ability to enhance maturation of human monocyte-derived DCs and exerts changes in their function, which is accompanied by constant antigen presentation to T cells, leading to uncontrolled T cell proliferation and disease progression (Jain et al., 2007). Furthermore, Tax induces the production of proinflammatory cytokines and chemokines involved in the activation of DCs (Jain et al., 2007). The production of CXCL10, CCL1, CCL3, and CCL4 is induced by Tax (Baba et al., 1996). CCL3 and CCL4 are among CC chemokines that attract DCs into the lymph nodes and play an important role in the effector phase of lymphocyte response and are capable of recruiting T cells with regulatory properties to the inflammation site (Sallusto et al., 1998b; Moser and Loetscher, 2001; Jain et al., 2007). CCL20/MIP-3α is a CC chemokine with a crucial role in the initiation of inflammation (Imaizumi et al., 2002).

It has been reported that Tax elevates the levels of CCL20 mRNA in HTLV-1 infected cell lines and human T cell lines. The specific receptor for this chemokine is CCR6, which is also increased in HTLV-1 infected cell lines suggesting an autocrine/paracrine mechanism for the development of HTLV-1-associated diseases (Baba et al., 1997; Imaizumi et al., 2002). Among various leukocyte subsets, CCR6 was detected and strongly up-regulated by IL-2 in lymphocytes (CD4^+^ and CD8^+^ T cells and B cells) but not in NK cells, monocytes, or granulocytes (Baba et al., 1997). CCL20 is responsible for chemo-attraction of effector/memory T cells and B cells through its receptor, CCR6 (Baba et al., 1997). Furthermore, CCL20 recruits immature DCs to the inflammation site and is crucial in the regulation of inflammation toward the skin and mucosal surfaces under homeostatic and inflammatory conditions, as well as in pathological conditions, including cancer and autoimmune disorders such as rheumatoid arthritis (Mantovani, 1999; Schutyser et al., 2003).

Tax induces CC chemokines including CCL5, CCL4 and, CCL3 in cultured peripheral blood mononuclear cells (PBMCs) from uninfected subjects (Barrios et al., 2011, 2014). These chemokines are known as HIV inhibitory factors and expression of CCL5, CCL4, CCL3, CCR5 ligands, is associated with the shift of HIV infection from macrophage to the T cell tropic (Moriuchi et al., 1998; Llano and Esté, 2005; Wang et al., 2017).

HBZ also plays a crucial role in the pathogenesis of ATL and HAM/TSP (Satou et al., 2006; Enose-Akahata et al., 2017). It has been shown that HBZ interacts with cAMP response element-binding protein (CREB) transcription factor, suppresses the Tax-mediated transactivation, and inhibits the canonical NF-kB pathway (Gaudray et al., 2002; Lemasson et al., 2007; Zhao et al., 2009). It has been reported that HBZ induces the production of CCL21 and CCR4 in HTLV-1 infected cells, therefore, supporting the proliferation and migration of infected T cells (Sugata et al., 2016; Naito et al., 2018).

HTLV-1 also incorporates viral protein Tax, and viral mRNAs including Tax, HBZ, and env, into exosomes derived from infected cells, which contribute to enhanced cellular FLICE-like inhibitory protein (cFLIP)-dependent survival of the recipient cell with induction of NF-kB, and resistance to apoptosis, by activation of AKT. Therefore, the exosomes derived from HTLV-1 infected cells might associate with HTLV-1 pathogenesis (Jaworski et al., 2014).

## Chemokines and Ham/Tsp Pathogenesis

In HAM/TSP patients, CD4^+^ CD25^+^ CCR4^+^ Foxp3^–^ T cells which serve as reservoirs for HTLV-1 (Yamano et al., 2009), express low levels of TGF-β and IL-10, while they express high levels of IFN-γ, thus, this pattern of cytokine production may contribute to disease pathogenesis (Yamano et al., 2009; Quaresma et al., 2016). High levels of the proinflammatory cytokines, chemokines, and MMP in HAM/TSP patients, is likely to be associated with HAM/TSP pathogenesis (Biddison et al., 1997; Lepoutre et al., 2009). The frequency of HTLV-1 specific CTLs is higher in HAM/TSP patients compared to ACs (Elovaara et al., 1993; Kubota et al., 1998). These CTLs express chemokine receptors with high migratory capacity (Bieganowska et al., 1999) involved in the migration of leukocytes to the site of inflammation and might play a crucial role in the pathogenesis of HAM/TSP. The neurological damages in CNS of HAM/TSP patients is caused by recognition of CD4^+^HTLV-1 infected cells in the CNS cells by specific CD8^+^ CTL, resulting in the production of inflammatory cytokines such as IFN-γ, TNF-α and MMP (Jacobson et al., 1990; Wucherpfennig et al., 1992; Kubota et al., 1994; Umehara et al., 1994, 1998). Furthermore, the interaction between HTLV-1-specific CTL and HTLV1-infected CD4+ T cells leads to apoptosis in surrounding neural cells such as oligodendrocytes and finally demyelination of the CNS (Ijichi et al., 1993; Hanon et al., 2000). Further studies have demonstrated that chemokines might contribute to the HAM/TSP pathogens and we summarize their results. According to the potential ability of chemokines to direct leukocyte trafficking, homing, and migration, in this study chemokines and their receptors are further classified based on their function.

### Trafficking

#### CXCL9 and CXCL10

CXCL9 and CXCL10 induce the recruitment of CXCR3 expressing cells including the CXCR3^+^ IFN-γ producing HTLV-1 infected CD4^+^ T cells that promote the production of CXCL10 by the astrocytes, creating a positive feedback loop that results in the CNS chronic inflammation (Futsch et al., 2018). In Multiple Sclerosis (MS), which is also a demyelinating neuroinflammatory disease, it is thought that CXCL9 and CXCL10 in the CSF are involved in the recruitment of CXCR3^+^ T cells into CNS and contribute to MS pathogenesis (Müller et al., 2010; Cheng and Chen, 2014; Vazirinejad et al., 2014; Koper et al., 2018).

CXCL9 and CXCL10 are presented in high levels in serum and CSF of patients with HAM/TSP and contribute to the pathogenesis of HAM/TSP (Sato et al., 2013; Guerra et al., 2018). Chaves et al. showed that the plasma levels of CXCL8 and CXCL9 were greater in HAM/TSP patients compared to carriers (Chaves et al., 2016). Moreover, a correlation was observed between plasma levels of CXCL8, CXCL9, and CXCL10 and proviral load, in which the HTLV-1 infected individuals with higher proviral loads had increased plasma levels of these chemokines (Chaves et al., 2016). CXCL10 in the CSF, as indicators of CNS inflammation, correlate with disease progression and has been suggested as a candidate for the prognosis of HAM/TSP development (Guerreiro et al., 2006; Sato et al., 2013; Lima et al., 2017). These markers might even be more potent than the proviral load for the prediction of disease progression in HTLV-1 infected individuals (Sato et al., 2013). Elevated CSF and serum levels of CXCL9 and CXCL10, compared to carriers, alongside low levels of CCL2 are considered as pivotal markers of HAM/TSP (Guerreiro et al., 2006; Sato et al., 2013; Starling et al., 2015; Lima et al., 2017). Amorim et al. (2014) showed that the production of both CXCL9 and CCL5 is elevated in HTLV-1 infected subjects compared with HCs. This high chemokine production might lead to increased inflammation in HTLV-1 infected individuals.

Sato et al. (2018b) showed that according to the concentrations of CSF CXCL10 and neopterin levels, HAM/TSP patients are classified into three groups based on disease activity and clinical progression rate. Further studies showed that low-dose oral prednisolone maintenance therapy decreased the CSF CXCL10 levels and is associated with good functional prognosis, suggesting CSF CXCL10 is a promising surrogate marker for HAM/TSP treatment (Tamaki et al., 2019).

#### CXCL8

CXCL8 might be associated with BBB disruption through the attraction of neutrophils within the CNS (Chaves et al., 2016). CXCL8 and its receptors, CXCR1 and CXCR2, are involved in the infiltration of leukocytes into the CNS (Almasi et al., 2013). CXCR1 and CXCR2 are expressed on neutrophils, monocytes, NK cells, CD4^+^ and CD8^+^T cells (Chuntharapai et al., 1994; Traves et al., 2004). CXCR2 has a role in inflammation and myelin disorders (Charo and Ransohoff, 2006). The levels of CXCR2 and its ligands are elevated in various CNS inflammatory conditions (Hosking et al., 2010). CXCR2 mRNA is highly expressed in HAM/TSP and CXCR2 CD8^+^ T cells have high frequencies in HAM/TSP patients. The high frequency of CXCR2 CD8^+^ T cells in HAM/TSP may contribute to demyelination and inflammation. However, CXCR1 CD8^+^ T cells are lower in HAM/TSP patients when compared to ACs. Since there is a negative correlation between proviral load and the percentage of T cells expressing CXCR1, it has been suggested that the high frequency of CXCR1 CD8^+^ T cells in ACs is associated with an efficient cell-mediated immune response that leads to limiting the spread of the virus (Rajaei et al., 2018). Thus, CXCL8, CXCL9, and CXCL10 are thought to be associated with HAM/TSP pathogenesis as well.

#### CCL2

CCL2 is thought to be associated with the migration of T cells and monocytes into the CNS of HAM/TSP patients. In HAM/TSP patients, CCL2, expressed in the CNS from vascular endothelial cells and mononuclear cells which have been infiltrated into the CNS, attracts T cells and monocytes into the CNS (Umehara et al., 1996). The lower levels of CCL2, which polarizes the immune response toward Th2 (Sallusto et al., 1998a), in HAM/TSP patients compared to HCs, also reflect a Th1 dominant immune response in HAM/TSP (Gu et al., 2000; Narikawa et al., 2005). It is likely that decreased levels of CCL2, which is associated with type two immune response, reflect the polarization of immune response toward a type 1 cytokine profile (Sallusto et al., 1998a; Gu et al., 2000). da Silva-Malta et al. reported that -46C/C polymorphism in the antigen receptor for chemokines (DARC) is associated with leukopenia, neutropenia and lower levels of CCL2, however, this polymorphism is not a risk factor for HAM/TSP (da Silva-Malta et al., 2017).

#### CCL20

The attraction of leukocytes to the site of inflammation may be conducted by CCL20/MIP-3α (Rossi et al., 1997). HTLV-1 infected individuals may develop inflammatory diseases according to CCL20 expression in HTLV-1 infected and uninfected T cells, which are induced by Tax through the NF-kB pathway. Since HTLV-1-infected T cells also express CCR6, the CCL20 receptor, an autocrine/paracrine mechanism may be involved in the pathogenesis of HTLV-1 associated diseases (Imaizumi et al., 2002; Rafatpanah et al., 2017). CCL20 is also a potent chemokine for the attraction of DCs into the CNS, suggesting infected DCs might play pivotal roles in HAM/TSP pathogenesis by induction of autoreactive T cells (Imaizumi et al., 2002).

#### CCL1

The role of CCL1 in the pathogenesis of experimental autoimmune encephalomyelitis (EAE) has been clearly investigated. It has been demonstrated that proteolipid protein-specific T-cell clones and production of the murine CCL1 homolog associates with EAE (Kuchroo et al., 1993; Teuscher et al., 1999). CCL1 is mainly produced by HTLV-1 infected cells of HAM/TSP patients in a Tax inducible and dependent manner and CCR8, the specific CCL1 receptor, is expressed on both HTLV-1 infected and uninfected T cells. The production of CCL1 is dependent on CREB and AP1 and the plasma levels of CCL1 are higher in HAM/TSP patients than the carriers and healthy subjects (Saito et al., 2017). The CCL1-CCR8 signaling axis may contribute to the pathogenesis of HAM/TSP, which might be suggested as a potential therapeutic target for immunotherapy of the disease (Saito et al., 2017).

#### CXCL11

CXCL11 or Interferon inducible T cell alpha chemoattractant (ITAC) enhances the chemotaxis of activated T cells and NK cells and its gene expression is induced by IFN-γ. CXCR3, the CXCL11 receptor, is also highly expressed on activated T cells, memory T cells, and NK cells and IFN-γ promotes its gene expression. It is also reported that CXCL11 is involved in the pathogenesis of cell mediated immunity (Müller et al., 2010). HAM/TSP patients have high levels of CXCL11 in plasma and CSF and the CSF CXCL11 can be used as a predictor of HAM/TSP development (Romanelli et al., 2018; Rosa et al., 2018).

### Homing

#### CCR4

CCR4^+^ T cells are selectively infected by HTLV-1 and converted into abnormal cells leading to the chronic inflammation in HAM/TSP patients (Araya et al., 2014; Yamano and Coler-Reilly, 2017). Elevated frequency of IFN-γ producing HTLV-1 infected CD4^+^CD25^+^CCR4^+^ T cells with proinflammatory features is found in HAM/TSP patients and this is associated with the disease pathogenesis (Araya et al., 2014; Yamano and Coler-Reilly, 2017). HTLV-1 induces transcriptional changes via Tax in infected T cells and downregulates the expression of FOXP3 in CD4^+^CD25^+^CCR4^+^ Tregs to lose expression of the transcription factor FOXP3 and increases the production of IFN-γ in these cells, which in turn leads to development of inflammation (Araya et al., 2014, 2015). The HTLV-1 Tax-expressing infected CD4^+^CD25^+^CCR4^+^ T cells exert changes in infected cells that may phenotypically become IFN-γ producing Th1-like cells, which probably enhance the inflammatory state (Araya et al., 2014, 2015). HBZ play a pivotal role in HAM/TSP by increasing the number of inducible Treg cells (iTreg) and induction of IFN-γ production (Yamamoto-Taguchi et al., 2013). Administration of Mogamulizumab/KW-0761, as a humanized anti-CCR4 monoclonal antibody (mAb), in HAM/TSP, has reduced the number of HTLV-1 infected cells and inflammatory markers. However, it was accompanied by adverse effects including rash, lymphopenia and leukopenia (Sato et al., 2018a).

### Migration

#### CCL3/MIP-1α and CCL4/MIP-1β

MIP can be selectively expressed and secreted in a *tax* transfected Jurkat cell line (Sharma and May, 1999). Elevated levels of MIP has been also demonstrated in the CSF of HAM/TSP patients (Miyagishi et al., 1995). HTLV-1-transformed T cells released CCL3/MIP-1α as a major monocyte chemoattractant, suggesting this molecule might play a pivotal role in the outcome of HTLV-1-related-diseases (Bertini et al., 1995).

In addition to Monocyte chemoattraction (Schall et al., 1990), CCL3 and CCL4 may attract CD8^+^ and CD4^+^ T cells (Taub et al., 1993). HTLV-1 specific CTLs produce CCL3 and CCL4, which may be related to inflammation, observed in HAM/TSP patients (Biddison et al., 1997).

## Chemokines and ATL Pathogenesis

ATL is an aggressive peripheral T cell neoplasm associated with HTLV-1 (Poiesz et al., 1981; Yoshida et al., 1982). ATL generally has a very poor prognosis and shorter overall survival (OS) compared to other peripheral T cell lymphoma (PTCL) (Vose et al., 2008). Clonal proliferation of HTLV-1 infected CD4^+^ T cells mediated by HTLV-1 viral factors, specifically Tax and HBZ promotes cellular transformation and leads to the development of ATL (Tanaka et al., 1990; Smith and Greene, 1991; Satou et al., 2006). Tax is able to stimulate cell proliferation in ATL and inhibits cell apoptosis (Yoshida, 2001; Matsuoka and Yasunaga, 2013; Mühleisen et al., 2014). In fact, Tax induces expression of anti-apoptotic proteins and genes which are involved in cell proliferation and consistently inactivates tumor suppressor proteins (Yoshida, 2001; Matsuoka and Yasunaga, 2013; Mühleisen et al., 2014). The increased cellular proliferation along with inhibited apoptosis results in prolonged cell survival and transformation of HTLV-1 infected cells (Yoshida, 2001). Since Tax is HTLV-1 specific major antigen that is recognized by CTLs, expression of Tax is lost in most of the ATL cases in order to escape host immune response (Kannagi et al., 1993). Despite Tax that is inactivated in most of the cases, HBZ is always expressed in all cases and plays an important role in leukemogenesis of HTLV-1 infected cells (Satou et al., 2006). In fact, Tax associates with the initiation of transformation, while HBZ is needed to maintain the transformation when Tax is silenced (Ma et al., 2016). HBZ also contributes to ATL oncogenesis by inhibition of apoptosis and supporting the proliferation and migration of ATL cells (Ma et al., 2013). The process of tissue infiltration of ATL cells and HTLV-1 infected T cells is likely to be regulated by chemokines, chemokine receptors, and adhesion molecules (Mori et al., 2000; Sugata et al., 2016). Here, we focus on chemokines and chemokine receptors involved in tissue infiltration of HTLV-1 infected and enhancement of proliferation, survival, and immortalization of ATL cells.

### Trafficking

#### CCL1/I-309 and CCR8

CCL1/I-309 is a chemokine with the ability of monocyte attraction (Miller and Krangel, 1992a, b). CCL1 observed in the supernatant of cultured ATL cells exerts anti-apoptotic effects against ATL cells (Ruckes et al., 2001). In addition to CCL1 production, ATL cells also express CCR8, the CCL1 chemokine receptor (Ruckes et al., 2001). The resistance of ATL cells to apoptosis might be attributed to the consequence of an autocrine loop between CCL1 and CCR8 (Ruckes et al., 2001).

#### CCL2/MCP-1

The elevated levels of monocyte chemoattractant protein 1 (MCP-1)/CCL2 mRNA in HTLV-1-infected T cell lines compared with uninfected cells have been reported (Mori et al., 2000). It is also shown that Tax induces the endogenous CCL2 through activation of the 5′ transcriptional regulatory region of the CCL2 gene in the human Jurkat T -cell line (Mori et al., 2000). Tax induces NF-κB binding to both CCL2 κB sites in order to transactivate the CCL2 gene via induction of NF-κB. Thus, the CCL2 gene regulation is disrupted by Tax and CCL2 is constitutively expressed in HTLV-1-infected cells (Mori et al., 2000). CCL2 also modulates the expression of leukocyte adhesion molecules and thus associates with tissue infiltration of leukocytes. Therefore, high levels of CCL2 expression in ATL cells may affect cell adhesion and tissue infiltration of ATL cells (Jiang et al., 1992). These findings might have important implications for our understanding of HTLV-1-associated diseases.

### Homing

#### CXCL12/SDF-1 α and CXCR4/CXCR7

The interaction between stromal cell-derived factor 1 (SDF1)/CXCL12 and CXCR4 has been identified to play a paramount role in the migration of ATL cells (Arai et al., 1998). Expression of CXCL12 in HTLV-1 infected T cell lines have been proven to be induced by Tax (Arai et al., 1998). It was also demonstrated that Tax enhances the CXCL12/CXCR4 axis in ATL cells (Twizere et al., 2007; Kawaguchi et al., 2009). Additionally, ADM3100 which serves as a CXCR4 antagonist prevents migration of human ATL cells and primary murine lymphoblastoid (pML) cells toward CXCL12 and also inhibits organ infiltration by lymphomatous cells in severe combined immunodeficient (SCID) mice (Kawaguchi et al., 2009). However, a study by Hasegawa et al. (2000) showed that the intensity of CXCR4 expression is regardless of ATL tissue infiltration.

Another chemokine receptor which is overexpressed in HTLV-1 infection is CXCR7 (Jin et al., 2009). CXCR7 serves as the CXCL12 receptor and is frequently expressed by various tumor cell lines and is also supposed to support cell survival and growth (Burns et al., 2006; Infantino et al., 2006; Wang et al., 2008). CXCR7 has been shown to be expressed on some human PBMCs including B cells, monocytes and DCs, while T cells mostly lack expression of CXCR7 (Burns et al., 2006; Infantino et al., 2006; Wang et al., 2008). Although CXCR7 is not expressed on T cells (Burns et al., 2006), a study by Jin et al. (2009) showed that CXCR7 expression on T cells is induced by Tax, suggesting contributes in enhancement of cell survival, growth, and immortalization of HTLV-1 infected cells. It has been shown that CCX754, the synthetic CXCR7 antagonist, inhibits the growth of HTLV-1 infected cells (Jin et al., 2009).

#### CXCL8

The CXCL8 mRNA expression has been detected in peripheral leukemic cells of ATL patients and in cultured HTLV-1-infected T cell lines, including HUT-102, MT-1, SALT-3, and SKT-1B (Mori et al., 1995). CXCL8 protein has also been measured in the culture medium of these cells and in the extracellular fluids of ATL patients (Mori et al., 1995). Based on these findings HTLV-1 tax gene may transactivate the CXCL8 gene through the NF-κB pathway in HTLV-1-infected cells. CXCL8 is rarely expressed in T cells, but its expression is increased in ATL cells and HTLV-1-infected cells, therefore, the potential role of CXCL8 in ATL pathogenesis remains to be determined (Mori et al., 1995).

#### CCL18

CCL18 is significantly expressed by DCs and mainly attracts naive T cells (Adema et al., 1997; Pivarcsi et al., 2004). Hence, it may critically be involved in the initiation of an immune response (Schutyser et al., 2005). A study conducted by Shimizu et al. reported that cases with HTLV-1 associated lymphadenitis type and Hodgkin’s like-type significantly express high levels of CCL18 compared to the non-specific lymphadenitis subjects (Shimizu et al., 2007). CCL18 can be associated with an efficient immune response in ATL patients through activation and attraction of leukocytes and thus plays a pivotal role in the initiation of the immune response against ATL cells (Shimizu et al., 2007).

### Migration

#### CCL25 and CCR9

The characteristic feature of ATL is organ infiltration by leukemic cells (Matsuoka, 2003). ATL cells from patients with GIT involvement have been found to express high levels of CCR9 which enables ATL cells to infiltrate into intestinal mucosa where CCL25, the only ligand for CCR9, is abundantly produced (Utsunomiya et al., 1988; Nagakubo et al., 2007). Nagakubo et al. (2007) reported that the expression of CCR9 on ATL cells is induced by Tax. Immunohistochemical studies have provided pieces of evidence that CCR9 is expressed on ATL cells that were infiltrated into GIT (Nagakubo et al., 2007). Additionally, according to the anti-apoptotic properties of CCL25 against CCR9 expressing cells, the CCL25/CCR9 interaction axis may be involved in ATL cells immortalization process (Youn et al., 2001; Nagakubo et al., 2007). However, the freshly isolated ATL cells have been reported to scarcely express CCR9 (Nagakubo et al., 2007).

#### CCL17/TARC, CCL22/MDC, and CCR4

CCR4 is highly expressed by most of ATL cells and is involved in the invasion of ATL cells into the skin tissues (Yoshie et al., 2002; Ishida et al., 2003; Hieshima et al., 2008). CCR4 is well established to be expressed on Th2 cells, Treg cells, and a fraction of memory T cells in which most of them are cutaneous lymphocyte antigen positive (CLA^+^) skin-homing memory T cells (Yoshie et al., 2001; Hieshima et al., 2008). Skin lesions are frequently observed in ATL and infiltration of ATL cells into skin can be directed by the interaction of CCR4 and its ligands, namely thymus and activation-regulated chemokine (TARC)/CCL17 as well as macrophage-derived chemokine (MDC)/CCL22 (Yoshie et al., 2002; Ishida et al., 2003; Shimauchi et al., 2005; Hiyoshi et al., 2015). Interestingly, a study by Shimauchi et al. (2005) demonstrated that CCR4^+^ ATL cells also produce CCR4 ligands, CCL17 and CCL22.

Tax induces CCL22 expression by infected T cells and facilitates the interaction between infected cells and CCR4^+^ uninfected cells. The efficient attraction of uninfected CCR4^+^ T cells into CCL22 expressing infected cells, alongside with formation of viral synapse results in HTLV-1 transmission, indicating that ATL cells and HTLV-1-infected T cells may originate preferentially from CCR4^+^ T cells (Hieshima et al., 2008). Additionally, the expression of CCR4 on HTLV-1 infected T cells is not Tax inducible. Therefore, the consistent CCR4 expression on ATL cells and HTLV-1 transformed T cells may be attributable to the perception that HTLV-1 preferentially infects CCR4^+^ T cells through the recruitment of CCR4^+^ T cells via CCL22 and/or due to some growth advantages of HTLV-1–infected CCR4^+^ T cells *in vivo* as well as *in vitro* (Yoshie et al., 2002). However, Sugata et al. (2016) showed that HBZ upregulates CCR4 expression in ATL and HTLV-1 infected cells and thus, migration and proliferation of those cells are increased, thus CCR4 may not be initially expressed on the infected cells (Percher et al., 2017). CD4^+^ FOXP3^+^ cells are observed at a high frequency in ATL patients (Toulza et al., 2009). The plasma levels of CCL22 in HTLV-1 infected individuals is elevated and correlates with the frequency of FOXP3^+^ cells that express CCR4. FOXP3^+^ cells play a dual role in ATL and they enhance the suppression of immune response and reduce disease progression (Toulza et al., 2010). CCL22, which is produced by HTLV-1 infected cells, may attract CD4^+^ CCR4^+^Foxp3^+^ T cells with regulatory functions (Toulza et al., 2010). These CD4^+^ CCR4^+^ Foxp3^+^ T cells may retard the progression of HTLV-1 associated diseases and also contribute to the immunosuppressive state in HTLV-1 infection (Toulza et al., 2008; Toulza et al., 2009, 2010).

Somatic mutations in the CCR4 gene occur in about 26% of ATL cases and might enhance cell migration toward CCL17 and CCL22 gradient which may contribute to ATL progression. Thus, CCR4 signaling plays an important role in ATL pathogenesis and it is a potential target for ATL therapy (Nakagawa et al., 2014). Several reports demonstrated that Mogamulizumab/KW-0761, as a humanized anti-CCR4 monoclonal antibody, has a potential efficacy against relapsed CCR4^+^ ATL cases and induces a long-term survival in cases who received Mogamulizumab monotherapy (Yamamoto et al., 2010; Ishida et al., 2017). According to previous studies, Mogamulizumab has increased antibody-dependent cell cytotoxicity (ADCC) of CCR4^+^ATL cells. This cytotoxicity is thought to be mainly mediated by NK cells (Ishida et al., 2012). Furthermore, a shortened form of Pseudomonas exotoxin, PE38, fused to CCL17, has been found to be able to kill HTLV-1-infected cells. This effect depends on the expression of both CCR4 and furin, a proprotein convertase, which are both elevated in HTLV-1-infected cells (Hiyoshi et al., 2015).

#### CCL19/ELC, CCL21/SLC and CCR7

Both EBI1-ligand chemokine (ELC)/CCL19 and secondary lymphoid-tissue chemokine (SLC)/CCL21 are well defined to play a role in the migration and homing of lymphocytes to lymphoid tissues via their common receptor, the CCR7 (Kozai et al., 2017). It has been shown that CCR7 is highly expressed on ATL cells from patients with lymphoid organ infiltration, thus it seems that CCR7 may be involved in the attraction of ATL cells into lymphoid tissues (Hasegawa et al., 2000). ATL can be further characterized by CCR7 expression within CD4^+^ CCR4^+^T cells, by which the CCR7^+^ phenotype refers to aggressive ATL, while CCR7^–^ phenotype refers to progressive indolent ATL (Kagdi et al., 2017). Gain-of-function mutations in CCR7 gene occurring in about 11% of ATL cases induce surface CCR7 expression and chemotaxis toward CCL19 and CCL21, and also enhances PI3K/AKT signaling, implicating the association of these activating mutations in the pathogenesis of ATL (Kataoka et al., 2015).

#### CCL5/RANTES

Regulated on activation, normal T cell expressed and secreted (RANTES)/CCL5 serves as a chemotactic factor for memory T cells that are produced by HTLV-1-infected cells and ATL cells in a Tax inducible manner (Mori et al., 2004). Although CCL5 is related to Th1 cells (Schrum et al., 1996), it is thought to be involved in ATL pathogenesis and tissue infiltration of infected cells, through CCR5 as its receptor (Mori et al., 2004). ATL and HTLV-1-infected cell lines produce high levels of CCL5 (Mori et al., 2004). Furthermore, HTLV-1 infected T cells have been found to particularly express the CCL5 gene as well as generate CCL5 (Mori et al., 2004). A recombinant vector containing the Tax-1 gene, *pCDNA3.1-TAX*, is also thought to be able to induce CCR5 expression in the K562 cell line (Haghnazari Sadaghiani et al., 2019). Furthermore, CCL5 is expressed by leukemic cells in peripheral blood and lymph nodes of ATL patients. It was shown that Tax induces the expression of CCL5 mRNA in a human Jurkat T cell line through NF-κB activation pathway (Mori et al., 2004). The increased expression of CCL5 by HTLV-1-infected T cells may contribute to pathophysiology of HTLV-1-associated diseases. However, its potential role remains to be determined.

#### CCL3/MIP-1α and CCL4/MIP-1β

CCL3/MIP-1α and CCL4/MIP-1β are highly produced in the culture supernatant and cytoplasmic fractions of tumor-infiltrating lymphocytes (Tanaka et al., 1998). It has been demonstrated that proinflammatory cytokines, including TNF-α, IL-1β, and IL-6 show increased levels in ATL cells. Tax enhances CCL3 and CCL4 production in ATL cells (Bertini et al., 1995; Tanaka et al., 1998), and it is thought that the elevated production of CCL3 and CCL4 chemokines in ATL cells might induce integrin-dependent adhesion of ATL cells to the endothelium and therefore may contribute to tissue infiltration of ATL cells. Pretreatment of ATL cells with antibodies against CCL3 and CCL4 led to a decrease of tissue infiltration of ATL cells through the reduction in integrin mediated adhesion (Tanaka et al., 1998).

#### CX3CL1/Fractalkine

Fractalkine or CX3CL1 is the only member of the CX3C chemokine subfamily (Zlotnik and Yoshie, 2000; Jones et al., 2010). CX3CL1 is found both in membrane-bound and soluble forms. The membrane-bound form acts as a chemoattractive factor and its expression is induced on endothelial cells by inflammatory cytokines (Bazan et al., 1997). The CX3CL1 receptor, CX3CR1, is expressed on a number of cells including cytotoxic effector lymphocytes like NK cells and cytotoxic effector T cells, mature monocytes/macrophages, and mucosal dendritic cells, which all are involved in elimination of cancer cells (Bazan et al., 1997; Imai et al., 2005). The gene expression of CX3CR1 is downregulated in ATL cells (Shimizu et al., 2007). In the Shimizu et al. study it was shown that the CX3CR1 expression in HTLV-1 associated subjects was lower than the non-specific lymphadenitis cases, suggesting HTLV-1 infection associates with host immunity through a reduction in the number of cytotoxic cells (Shimizu et al., 2007). These results proposed that downregulation of CX3CR1 plays an important part in immune response against the ATL cells.

## Chemokines and Other HTLV-1-Associated Diseases

It is well documented that ATL is associated with severe immunosuppression and patients with ATL are prone to opportunistic infections (Uchiyama et al., 1977; Bunn et al., 1983; Shimoyama and Group, 1991). However, the immune response controlling infection in HAM/TSP patients may become more harmful and participate in HAM/TSP development (Asquith and Bangham, 2008). Changes in immune response may also be related to other HTLV-1 associated diseases. Pieces of evidence indicate that different types of skin lesions observed in HTLV-1 carriers may be associated with a distinct imbalance of inflammatory response. The serum levels of CCL5, CXCL8, CXCL9, and CXCL10 have shown alterations in HTLV-1 infected individuals with or without skin lesions. It has been reported that HTLV-1 infected cases with autoimmune lesions have elevated levels of CCL5 and CXCL8 compared to those with infectious lesions. CXCL10 level has also been found to be elevated in HTLV-1 infected subjects with autoimmune lesions compared to the cases without lesions (Coelho-dos-Reis et al., 2013). Infection of lung epithelial cells by HTLV-1 was tested by co-culturing A549 alveolar and NCIH292 tracheal epithelial cell lines with MT-2 cell line which is an HTLV-1 infected T cell line. The HTLV-1 infected lung epithelial cells produce inflammatory chemokines such as CCL2, CCL5, and CCL20 (Teruya et al., 2008), which are all able to contribute to the pathogenesis of HTLV-1 associated pulmonary disorders (Teruya et al., 2008).

CCL5 is highly chemotactic for memory T cells and monocytes (Schall et al., 1990). This chemokine is expressed in HTLV-1-infected cell lines and primary ATL cells and may have a role in tissue infiltration (Mori et al., 2004). Monocytes have been suggested to be involved in immune regulation and progression of HTLV-1 associated diseases (de Castro-Amarante et al., 2016). HTLV-1 provirus has been detected in all three subsets of monocyte (classical, intermediate, non-classical) and found to have a profound impact on surface receptors, migratory function, and the frequency of all monocyte subsets. In HTLV-1 infected subjects, classical monocytes express high levels of CCR5, CXCR3 and CX3CR1 and a correlation has been observed between the proviral load and the migratory capacity of classical monocytes toward CCL2, CCL5, and CX3CL1, suggesting that migration of monocyte into inflammatory sites is related with persistence of HTLV-1 infection and progression of HTLV-1 associated inflammatory diseases, such as Myositis, Uveitis, and HAM/TSP (de Castro-Amarante et al., 2016).

The levels of some chemokines including CXCL9 and CXCL10 are undetectable in neutrophils from HTLV-1 infected and uninfected subjects (Bezerra et al., 2011). Furthermore, although neutrophils are of the main sources of CXCL8 and CCL4, these two chemokines have similar production in neutrophils from both infected and uninfected subjects, indicating that neutrophils effector functions are not impaired in these two groups (Bezerra et al., 2011). Pulmonary disorders can develop in ACs as well as HTLV-1 infected patients (Seki et al., 1999; Yamazato et al., 2003; Yamamoto et al., 2004; Nakayama et al., 2013). High levels of CCL3 and CCL5 in bronchoalveolar lavage fluid (BALF) have been detected in HTLV-1 carriers (Seki et al., 1999; Yamazato et al., 2003). Since CCL3 and CCL5 are involved in the attraction of T cells and memory T cells, high levels of these β-chemokines in HTLV-1 carriers may play a role in developing pulmonary disorders (Seki et al., 1999). It has been reported that in HTLV-1 positive cryptogenic fibrosing alveolitis (CFA) patients, a chronic inflammatory interstitial lung disease, the BALF levels of CCL3 (Taub et al., 1996; Matsuyama et al., 2003) and CXCL10, T cell chemoattractants and activators (Loetscher et al., 1996; Matsuyama et al., 2003), are higher than those of without HTLV-1 infection. Furthermore, CCL3 and CXCL10 BALF levels were associated with an increased number of activated T cells. Moreover, HTLV-1 infection may also contribute in the development of CFA (Matsuyama et al., 2003).

HTLV-1 infected individuals with overactive bladder, have similar serum concentrations of CXCL9 and CXCL10 compared to the carriers (Santos et al., 2012). Adding exogenous IL-10 and TGF-β to the cell cultures from HTLV-1 overactive bladder patients decreased the production of IFN-γ (Santos et al., 2012). The proviral load and levels of some immunological factors such as TNF-α and IL-17 in overactive bladder patients are similar to HAM/TSP patients. According to these observations, the overactive bladder is a common condition between carriers and HAM/TSP patients, and it was proposed that patients with overactive bladder may further develop HAM/TSP (Santos et al., 2012). Supplementary Table 1 represents the chemokines and chemokine receptors associated with HTLV-1 pathogenesis.

The following issues influence the limitation of the present review including: omission of relevant research, the authors’ point of view, and misconception in the translation of data from the primary literature. Controversial results of the study may have been observed due to an analysis of chemokine levels in serum/plasma, CSF and supernatant of HTLV-1 cell lines and PBMCs culture, sample size, disease duration, clinical status and heterogeneity of patients and HTLV-1 carriers. To the best of our knowledge, this review has been one of the first attempts to thoroughly explain the role of chemokines and their receptors in HTLV-1 infection; as such, a wide range of data regarding the chemokines and receptors are a strength of the study.

In this study, the status of chemokines and chemokine receptors was discussed in HTLV-1 infection focusing on two major diseases associated with the virus: ATL and HAM/TSP. According to the available studies reviewed in this work, it is clear that chemokines play a critical role in inflammation and regulating the trafficking, migration, and tissue infiltration of leukocytes, and the dysregulation of various chemokines could play an important role in the pathophysiology of HTLV-1 associated disease. Clinical studies in HAM/TSP and ATL patients have been provided promising results to be hopeful to develop and recommend more effective inhibitors of chemokines and their receptors. Therefore, development of chemokine-directed therapeutic strategies for targeting leukocyte recruitment pathways could be a promising therapeutic approach for HTLV-1 infection.

## Conclusion

In this review, we have discussed the potential role of chemokines and their receptors in the outcome of HTLV-1 associated diseases. A great number of chemokines and their respective receptors have been implicated in the migration of HTLV-1 infected cells into CNS and skin lesions are a vital aspect in pathogenesis of HTLV-1-associated disease. Elevation of chemokines levels in serum and CSF in HTLV-1 infection has been considered as prognostic biomarkers and a promising surrogate marker for HAM/TSP treatment. The complexity of the chemokine network is perplexing. Thus, further investigation is required to target chemokine network that could open up a new door to therapy and prophylaxis against HAM/TSP and ATL.

## Author Contributions

HR designed the study and wrote the manuscript. RZ designed and drafted the manuscript. MM drafted and wrote the manuscript. AM and GH revised the manuscript. ZV drafted the manuscript.

## Conflict of Interest

The authors declare that the research was conducted in the absence of any commercial or financial relationships that could be construed as a potential conflict of interest.

## Abbreviations

ACs: asymptomatic carriers
BALF: bronchoalveolar lavage fluid
CFA: cryptogenic fibrosing alveolitis
CLA^+^: cutaneous lymphocyte antigen positive
CTCL: cutaneous T cell lymphoma
CTLs: cytotoxic T lymphocytes
ELC: EBI1 ligand chemokine
FOXP3: Forkhead Box P3
GIT: gastrointestinal tract
GPCRs: G Protein–Coupled Receptors
HBZ: HTLV-1 bZIP factor
HCs: healthy controls
IL: interleukin
IP-10: IFN- γ inducible Protein-10
LFA-1: lymphocyte function-associated antigen 1
MCP-1: monocyte chemotactic protein-1
MDC: macrophage-derived chemokine
MIP: macrophage inflammatory protein
pML: primary murine lymphoblastoid
RANTES: regulated on activation normal T expressed and secreted
SCID: severe combined immunodeficiency
SDF-1 α: Stromal cell-Derived Factor-1
SLC: secondary lymphoid-tissue chemokine
TARC: thymus activation-regulated chemokine.

## References

[B1] Adema G. J. Hartgers F. Verstraten R. de Vries E. Marland G. Menon S. (1997). A dendritic-cell-derived C-C chemokine that preferentially attracts naive T cells. *Nature* 387 713–717. 10.1038/42716 9192897

[B2] Afonso P. V. Ozden S. Cumont M. C. Seilhean D. Cartier L. Rezaie P. (2008). Alteration of blood-brain barrier integrity by retroviral infection. *PLoS Pathog.* 4:e1000205. 10.1371/journal.ppat.1000205 19008946 PMC2575404

[B3] Afonso P. V. Ozden S. Prevost M.-C. Schmitt C. Seilhean D. Weksler B. (2007). Human blood-brain barrier disruption by retroviral-infected lymphocytes: role of myosin light chain kinase in endothelial tight-junction disorganization. *J. Immunol.* 179 2576–2583. 10.4049/jimmunol.179.4.2576 17675520

[B4] Ahuja J. Lepoutre V. Wigdahl B. Khan Z. K. Jain P. (2007). Induction of pro-inflammatory cytokines by human T-cell leukemia virus type-1 Tax protein as determined by multiplexed cytokine protein array analyses of human dendritic cells. *Biomed. Pharmacother.* 61 201–208. 10.1016/j.biopha.2007.02.006 17391906 PMC2043123

[B5] Almasi S. Aliparasti M. R. Farhoudi M. Babaloo Z. Baradaran B. Zamani F. (2013). Quantitative evaluation of CXCL8 and its receptors (CXCR1 and CXCR2) gene expression in Iranian patients with multiple sclerosis. *Immunol. Investig.* 42 737–748. 10.3109/08820139.2013.812652 23876169

[B6] Amorim C. F. Souza A. S. Diniz A. G. Carvalho N. B. Santos S. B. Carvalho E. M. (2014). Functional activity of monocytes and macrophages in HTLV-1 infected subjects. *PLoS Negl. Trop. Dis.* 8:e3399. 10.1371/journal.pntd.0003399 25521499 PMC4270688

[B7] Ando H. Sato T. Tomaru U. Yoshida M. Utsunomiya A. Yamauchi J. (2013). Positive feedback loop via astrocytes causes chronic inflammation in virus-associated myelopathy. *Brain* 136 2876–2887. 10.1093/brain/awt183 23892452

[B8] Apolinario A. Majano P. L. Alvarez-Pérez E. Saez A. Lozano C. Vargas J. (2002). Increased expression of T cell chemokines and their receptors in chronic hepatitis C: relationship with the histological activity of liver disease. *Am. J. Gastroenterol.* 97 2861–2870. 10.1111/j.1572-0241.2002.07054.x 12425561

[B9] Arai M. Ohashi T. Tsukahara T. Murakami T. Hori T. Uchiyama T. (1998). Human T-cell leukemia virus type 1 Tax protein induces the expression of lymphocyte chemoattractant SDF-1/PBSF. *Virology* 241 298–303. 10.1006/viro.1997.8968 9499804

[B10] Araya N. Sato T. Ando H. Tomaru U. Yoshida M. Coler-Reilly A. (2014). HTLV-1 induces a Th1-like state in CD4+CCR4+ T cells. *J. Clin. Invest.* 124 3431–3442. 10.1172/jci75250 24960164 PMC4109535

[B11] Araya N. Sato T. Tomaru U. Coler-Reilly A. Yagishita N. Yamauchi J. (2015). HTLV-1 Tax induces Th1 master regulator T-bet and thus IFN-γ in CD4+CCR4+ T-cells of virus-associated myelopathy patients. *Retrovirology* 12:44 10.1186/1742-4690-12-s1-p44

[B12] Asquith B. Bangham C. R. (2008). How does HTLV-I persist despite a strong cell-mediated immune response? *Trends Immunol.* 29 4–11. 10.1016/j.it.2007.09.006 18042431 PMC6066088

[B13] Baba M. Imai T. Nishimura M. Kakizaki M. Takagi S. Hieshima K. (1997). Identification of CCR6, the specific receptor for a novel lymphocyte-directed CC chemokine LARC. *J. Biol. Chem.* 272 14893–14898. 10.1074/jbc.272.23.14893 9169459

[B14] Baba M. Imai T. Yoshida T. Yoshie O. (1996). Constitutive expression of various chemokine genes in human T-cell lines infected with human T-cell leukemia virus type 1: role of the viral transactivator Tax. *Int. J. Cancer* 66 124–129. 10.1002/(sici)1097-0215(19960328)66:1¡124::aid-ijc21¿3.0.co;2-c 8608955

[B15] Banerjee P. Feuer G. Barker E. (2007). Human T-cell leukemia virus type 1 (HTLV-1) p12I down-modulates ICAM-1 and -2 and reduces adherence of natural killer cells, thereby protecting HTLV-1-infected primary CD4+ T cells from autologous natural killer cell-mediated cytotoxicity despite the reduction of major histocompatibility complex class I molecules on infected cells. *J. Virol.* 81 9707–9717. 10.1128/jvi.00887-07 17609265 PMC2045425

[B16] Bangham C. R. Araujo A. Yamano Y. Taylor G. P. (2015). HTLV-1-associated myelopathy/tropical spastic paraparesis. *Nat. Rev. Dis. Primers.* 1:15012. 10.1038/nrdp.2015.12 27188208

[B17] Barrios C. S. Abuerreish M. Lairmore M. D. Castillo L. Giam C. Z. Beilke M. A. (2011). Recombinant human T-cell leukemia virus types 1 and 2 Tax proteins induce high levels of CC-chemokines and downregulate CCR5 in human peripheral blood mononuclear cells. *Viral. Immunol.* 24 429–439. 10.1089/vim.2011.0037 22111594 PMC3234705

[B18] Barrios C. S. Castillo L. Zhi H. Giam C. Z. Beilke M. (2014). Human T cell leukaemia virus type 2 tax protein mediates CC-chemokine expression in peripheral blood mononuclear cells via the nuclear factor kappa B canonical pathway. *Clin. Exp. Immunol.* 175 92–103. 10.1111/cei.12213 24116893 PMC3898558

[B19] Bazan J. F. Bacon K. B. Hardiman G. Wang W. Soo K. Rossi D. (1997). A new class of membrane-bound chemokine with a CX3C motif. *Nature* 385 640–644. 10.1038/385640a0 9024663

[B20] Bertini R. Luini W. Sozzani S. Bottazzi B. Ruggiero P. Boraschi D. (1995). Identification of MIP-1α/LD78 as a monocyte chemoattractant released by the HTLV-I-transformed cell line MT4. *AIDS Res. Hum. Retroviruses* 11 155–160. 10.1089/aid.1995.11.155 7537510

[B21] Bex F. Yin M.-J. Burny A. Gaynor R. B. (1998). Differential transcriptional activation by human T-cell leukemia virus type 1 Tax mutants is mediated by distinct interactions with CREB binding protein and p300. *Mol. Cell. Bio.* 18 2392–2405. 10.1128/mcb.18.4.2392 9528808 PMC121497

[B22] Bezerra C. A. Cardoso T. M. Giudice A. Porto A. F. Santos S. B. Carvalho E. M. (2011). Evaluation of the microbicidal activity and cytokines/chemokines profile released by neutrophils from HTLV-1-Infected Individuals. *Scand. J. Immunol.* 74 310–317. 10.1111/j.1365-3083.2011.02579.x 21595736 PMC3153872

[B23] Biddison W. E. Kubota R. Kawanishi T. Taub D. D. Cruikshank W. W. Center D. M. (1997). Human T cell leukemia virus type I (HTLV-I)-specific CD8+ CTL clones from patients with HTLV-I-associated neurologic disease secrete proinflammatory cytokines, chemokines, and matrix metalloproteinase. *J. Immunol.* 159 2018–2025. 9257869

[B24] Bieganowska K. Hollsberg P. Buckle G. J. Lim D. G. Greten T. F. Schneck J. (1999). Direct analysis of viral-specific CD8+ T cells with soluble HLA-A2/Tax11-19 tetramer complexes in patients with human T cell lymphotropic virus-associated myelopathy. *J. Immunol.* 162 1765–1771. 9973440

[B25] Blanchet X. Langer M. Weber C. Koenen R. R. von Hundelshausen P. (2012). Touch of chemokines. *Front. Immunol.* 3:175. 10.3389/fimmu.2012.00175 22807925 PMC3394994

[B26] Bonville C. Rosenberg H. Domachowske J. (1999). Macrophage inflammatory protein–1α and RANTES are present in nasal secretions during ongoing upper respiratory tract infection. *Pediatr. Allergy Immunol.* 10 39–44. 10.1034/j.1399-3038.1999.101005.x 10410916

[B27] Bunn P. A. Jr. Schechter G. P. Jaffe E. Blayney D. Young R. C. Matthews M. J. (1983). Clinical course of retrovirus-associated adult T-cell lymphoma in the United States. *N. Engl. J. Med* 309 257–264. 10.1056/nejm198308043090501 6602943

[B28] Burns J. M. Summers B. C. Wang Y. Melikian A. Berahovich R. Miao Z. (2006). A novel chemokine receptor for SDF-1 and I-TAC involved in cell survival, cell adhesion, and tumor development. *J. Exp. Med.* 203 2201–2213. 10.1084/jem.20052144 16940167 PMC2118398

[B29] Cavanagh M.-H. Landry S. Audet B. Arpin-André C. Hivin P. Paré M.-E. (2006). HTLV-I antisense transcripts initiating in the 3′LTR are alternatively spliced and polyadenylated. *Retrovirology* 3 15–15. 10.1186/1742-4690-3-15 16512901 PMC1459196

[B30] Charo I. F. Ransohoff R. M. (2006). The many roles of chemokines and chemokine receptors in inflammation. *N. Engl. J. Med.* 354 610–621. 10.1056/NEJMra052723 16467548

[B31] Chaves D. G. Sales C. C. de Cassia Goncalves P. da Silva-Malta M. C. Romanelli L. C. Ribas J. G. (2016). Plasmatic proinflammatory chemokines levels are tricky markers to monitoring HTLV-1 carriers. *J. Med. Virol.* 88 1438–1447. 10.1002/jmv.24481 26800845

[B32] Cheng W. Chen G. (2014). Chemokines and chemokine receptors in multiple sclerosis. *Med. Inflamm.* 2014:659206. 10.1155/2014/659206 24639600 PMC3930130

[B33] Chuntharapai A. Lee J. Hebert C. A. Kim K. J. (1994). Monoclonal antibodies detect different distribution patterns of IL-8 receptor A and IL-8 receptor B on human peripheral blood leukocytes. *J. Immunol.* 153 5682–5688. 7527448

[B34] Coelho-dos-Reis J. G. A. Passos L. Duarte M. C. Araújo M. G. Campi-Azevedo A. C. Teixeira-Carvalho A. (2013). Immunological profile of HTLV-1-infected patients associated with infectious or autoimmune dermatological disorders. *PLoS Negl. Trop. Dis.* 7:e2328. 10.1371/journal.pntd.0002328 23936564 PMC3723575

[B35] Currer R. Van Duyne R. Jaworski E. Guendel I. Sampey G. Das R. (2012). HTLV tax: a fascinating multifunctional co-regulator of viral and cellular pathways. *Front. Microbiol.* 3:406. 10.3389/fmicb.2012.00406 23226145 PMC3510432

[B36] Cyster J. G. (1999). Chemokines and cell migration in secondary lymphoid organs. *Science* 286 2098–2102. 10.1126/science.286.5447.2098 10617422

[B37] da Silva-Malta M. C. F. Sales C. C. Guimaraes J. C. de Cassia Goncalves P. Chaves D. G. Santos H. C. (2017). The Duffy null genotype is associated with a lower level of CCL2, leukocytes and neutrophil count but not with the clinical outcome of HTLV-1 infection. *J. Med. Microbiol.* 66 1207–1216. 10.1099/jmm.0.000539 28771137

[B38] Dahm T. Rudolph H. Schwerk C. Schroten H. Tenenbaum T. (2016). Neuroinvasion and Inflammation in Viral Central Nervous System Infections. *Mediators Inflamm.* 2016:8562805. 10.1155/2016/8562805 27313404 PMC4897715

[B39] Dawson T. C. Beck M. A. Kuziel W. A. Henderson F. Maeda N. (2000). Contrasting effects of CCR5 and CCR2 deficiency in the pulmonary inflammatory response to influenza A virus. *Am. J. Pathol.* 156 1951–1959. 10.1016/s0002-9440(10)65068-7 10854218 PMC1850091

[B40] de Castro-Amarante M. F. Pise-Masison C. A. McKinnon K. Parks R. W. Galli V. Omsland M. (2016). Human T Cell leukemia virus type 1 infection of the three monocyte subsets contributes to viral burden in humans. *J. Virol.* 90 2195–2207. 10.1128/JVI.02735-15 26608313 PMC4810698

[B41] Domachowske J. B. Bonville C. A. Easton A. J. Rosenberg H. F. (2002). Differential expression of proinflammatory cytokine genes in vivo in response to pathogenic and nonpathogenic pneumovirus infections. *J. Infect. Dis.* 186 8–14. 10.1086/341082 12089656

[B42] Elovaara I. Koenig S. Brewah A. Y. Woods R. Lehky T. Jacobson S. (1993). High human T cell lymphotropic virus type 1 (HTLV-1)-specific precursor cytotoxic T lymphocyte frequencies in patients with HTLV-1-associated neurological disease. *J. Exp. Med.* 177 1567–1573. 10.1084/jem.177.6.1567 8496677 PMC2191033

[B43] El-Sabban M. E. Merhi R. A. Haidar H. A. Arnulf B. Khoury H. Basbous J. (2002). Human T-cell lymphotropic virus type 1-transformed cells induce angiogenesis and establish functional gap junctions with endothelial cells. *Blood* 99 3383–3389. 10.1182/blood.v99.9.3383 11964307

[B44] Enose-Akahata Y. Vellucci A. Jacobson S. (2017). Role of HTLV-1 Tax and HBZ in the Pathogenesis of HAM/TSP. *Front. Microbiol.* 8:2563. 10.3389/fmicb.2017.02563 29312243 PMC5742587

[B45] Fujii M. Chuhjo T. Minamino T. Masaaki N. Miyamoto K.-I. Seiki M. (1995). Identification of the Tax interaction region of serum response factor that mediates the aberrant induction of immediate early genes through CArG boxes by HTLV-I Tax. *Oncogene* 11 7–14. 7624133

[B46] Fujii M. Iwai K. Oie M. Fukushi M. Yamamoto N. Kannagi M. (2000). Activation of oncogenic transcription factor AP-1 in T cells infected with human T cell leukemia virus type 1. *AIDS Res. Hum. Retroviruses* 16 1603–1606. 10.1089/08892220050193029 11080798

[B47] Fujii M. Tsuchiya H. Chuhjo T. Akizawa T. Seiki M. (1992). Interaction of HTLV-1 Tax1 with p67SRF causes the aberrant induction of cellular immediate early genes through CArG boxes. *Genes Dev.* 6 2066–2076. 10.1101/gad.6.11.2066 1427072

[B48] Furukawa Y. Yamashita M. Usuku K. Izumo S. Nakagawa M. Osame M. (2000). Phylogenetic subgroups of human t cell lymphotropic virus (HTLV) type i in the tax gene and their association with different risks for HTLV-I—associated myelopathy/tropical spastic paraparesis. *J. Infect. Dis.* 182 1343–1349. 10.1086/315897 11010842

[B49] Futsch N. Prates G. Mahieux R. Casseb J. Dutartre H. (2018). Cytokine networks dysregulation during HTLV-1 Infection and Associated Diseases. *Viruses* 10:691. 10.3390/v10120691 30563084 PMC6315340

[B50] Gaudray G. Gachon F. Basbous J. Biard-Piechaczyk M. Devaux C. Mesnard J. M. (2002). The complementary strand of the human T-cell leukemia virus type 1 RNA genome encodes a bZIP transcription factor that down-regulates viral transcription. *J. Virol.* 76 12813–12822. 10.1128/jvi.76.24.12813-12822.2002 12438606 PMC136662

[B51] Gessain A. Barin F. Vernant J. C. Gout O. Maurs L. Calender A. (1985). Antibodies to human T-lymphotropic virus type-I in patients with tropical spastic paraparesis. *Lancet* 2 407–410. 10.1016/s0140-6736(85)92734-5 2863442

[B52] Giraudon P. Bernard A. Malcus C. Dufay N. Desgranges C. Belin M. F. (1995). Retroviral Infection (HTLV-I) induces cytokine-regulated immunomodulation and cytotoxicity of medulloblastoma cells. *J. Neuropathol. Exp. Neurol.* 54 165–174. 10.1097/00005072-199503000-00003 7876886

[B53] Green J. E. Hinrichs S. H. Vogel J. Jay G. (1989). Exocrinopathy resembling Sjogren’s syndrome in HTLV-1 tax transgenic mice. *Nature* 341 72–74. 10.1038/341072a0 2788824

[B54] Gu L. Tseng S. Horner R. M. Tam C. Loda M. Rollins B. J. (2000). Control of TH2 polarization by the chemokine monocyte chemoattractant protein-1. *Nature* 404 407–411. 10.1038/35006097 10746730

[B55] Guerra M. Luna T. Souza A. Amorim C. Carvalho N. B. Carvalho L. (2018). Local and systemic production of proinflammatory chemokines in the pathogenesis of HAM/TSP. *Cell Immunol.* 334 70–77. 10.1016/j.cellimm.2018.09.009 30473006

[B56] Guerreiro J. Santos S. Morgan D. Porto A. Muniz A. Ho J. (2006). Levels of serum chemokines discriminate clinical myelopathy associated with human T lymphotropic virus type 1 (HTLV-1)/tropical spastic paraparesis (HAM/TSP) disease from HTLV-1 carrier state. *Clin. Exp. Immunol.* 145 296–301. 10.1111/j.1365-2249.2006.03150.x 16879249 PMC1809672

[B57] Haeberle H. A. Kuziel W. A. Dieterich H.-J. Casola A. Gatalica Z. Garofalo R. P. (2001). Inducible expression of inflammatory chemokines in respiratory syncytial virus-infected mice: role of MIP-1α in lung pathology. *J. Virol.* 75 878–890. 10.1128/jvi.75.2.878-890.2001 11134301 PMC113984

[B58] Haghnazari Sadaghiani N. Pirayeshfard L. Aghaie A. Sharifi Z. (2019). The Effect of TAX-1 gene of human T-cell leukemia virus Type-1 on the Expression of CCR5 in K562 Cell Line. *Avicenna J. Med. Biotechnol.* 11 67–71. 30800245 PMC6359701

[B59] Hall W. W. Liu C. R. Schneewind O. Takahashi H. Kaplan M. H. Roupe G. (1991). Deleted HTLV-I provirus in blood and cutaneous lesions of patients with mycosis fungoides. *Science* 253 317–320. 10.1126/science.1857968 1857968

[B60] Hanon E. Hall S. Taylor G. P. Saito M. Davis R. Tanaka Y. (2000). Abundant tax protein expression in CD4+ T cells infected with human T-cell lymphotropic virus type I (HTLV-I) is prevented by cytotoxic T lymphocytes. *Blood* 95 1386–1392. 10.1182/blood.v95.4.1386.004k22_1386_1392 10666215

[B61] Hasegawa H. Nomura T. Kohno M. Tateishi N. Suzuki Y. Maeda N. (2000). Increased chemokine receptor CCR7/EBI1 expression enhances the infiltration of lymphoid organs by adult T-cell leukaemia cells. *Blood* 95 30–38. 10.1182/blood.v95.1.30.001k09_30_38 10607681

[B62] Hieshima K. Nagakubo D. Nakayama T. Shirakawa A.-K. Jin Z. Yoshie O. (2008). Tax-inducible production of CC chemokine ligand 22 by human T cell leukemia virus type 1 (HTLV-1)-infected T cells promotes preferential transmission of HTLV-1 to CCR4-expressing CD4+ T cells. *J. Immunol.* 180 931–939. 10.4049/jimmunol.180.2.931 18178833

[B63] Hiyoshi M. Okuma K. Tateyama S. Takizawa K. Saito M. Kuramitsu M. (2015). Furin-dependent CCL17-fused recombinant toxin controls HTLV-1 infection by targeting and eliminating infected CCR4-expressing cells in vitro and in vivo. *Retrovirology* 12:73. 10.1186/s12977-015-0199-8 26289727 PMC4545545

[B64] Hosking M. P. Tirotta E. Ransohoff R. M. Lane T. E. (2010). CXCR2 signaling protects oligodendrocytes and restricts demyelination in a mouse model of viral-induced demyelination. *PloS One* 5:e11340. 10.1371/journal.pone.0011340 20596532 PMC2893165

[B65] Ijichi S. Izumo S. Eiraku N. Machigashira K. Kubota R. Nagai M. (1993). An autoaggressive process against bystander tissues in HTLV-I-infected individuals: a possible pathomechanism of HAM/TSP. *Med. Hypotheses* 41 542–547. 10.1016/0306-9877(93)90111-3 8183132

[B66] Imai T. Nishimura M. Nanki T. Umehara H. (2005). [Fractalkine and inflammatory diseases]. *Nihon Rinsho Meneki Gakkai Kaishi* 28 131–139. 10.2177/jsci.28.131 15997176

[B67] Imaizumi Y. Sugita S. Yamamoto K. Imanishi D. Kohno T. Tomonaga M. (2002). Human T cell leukemia virus type-I Tax activates human macrophage inflammatory protein-3α/CCL20 gene transcription via the NF-κB pathway. *Int. Immunol.* 14 147–155. 10.1093/intimm/14.2.147 11809734

[B68] Inagaki A. Ishida T. Ishii T. Komatsu H. Iida S. Ding J. (2006). Clinical significance of serum Th1-, Th2- and regulatory T cells-associated cytokines in adult T-cell leukemia/lymphoma: high interleukin-5 and -10 levels are significant unfavorable prognostic factors. *Int. J Cancer* 118 3054–3061. 10.1002/ijc.21688 16425276

[B69] Infantino S. Moepps B. Thelen M. (2006). Expression and regulation of the orphan receptor RDC1 and its putative ligand in human dendritic and B cells. *J. Immuno.* 176 2197–2207. 10.4049/jimmunol.176.4.2197 16455976

[B70] Ishida T. Joh T. Uike N. Yamamoto K. Utsunomiya A. Yoshida S. (2012). Defucosylated anti-CCR4 monoclonal antibody (KW-0761) for relapsed adult T-cell leukemia-lymphoma: a multicenter phase II study. *J. Clin. Oncol.* 30 837–842. 10.1200/jco.2011.37.3472 22312108

[B71] Ishida T. Utsunomiya A. Iida S. Inagaki H. Takatsuka Y. Kusumoto S. (2003). Clinical significance of CCR4 expression in adult T-cell leukemia/lymphoma: its close association with skin involvement and unfavorable outcome. *Clin. Cancer Res.* 9(10 Pt 1), 3625–3634. 14506150

[B72] Ishida T. Utsunomiya A. Jo T. Yamamoto K. Kato K. Yoshida S. (2017). Mogamulizumab for relapsed adult T-cell leukemia-lymphoma: updated follow-up analysis of phase I and II studies. *Cancer Sci.* 108 2022–2029. 10.1111/cas.13343 28776876 PMC5623751

[B73] Ishida Y. Gao J.-L. Murphy P. M. (2008). Chemokine receptor CX3CR1 mediates skin wound healing by promoting macrophage and fibroblast accumulation and function. *J. Immunol.* 180 569–579. 10.4049/jimmunol.180.1.569 18097059

[B74] Jacobson S. Shida H. McFarlin D. E. Fauci A. S. Koenig S. (1990). Circulating CD8+ cytotoxic T lymphocytes specific for HTLV-I pX in patients with HTLV-I associated neurological disease. *Nature* 348 245–248. 10.1038/348245a0 2146511

[B75] Jain P. Ahuja J. Khan Z. K. Shimizu S. Meucci O. Jennings S. R. (2007). Modulation of dendritic cell maturation and function by the Tax protein of human T cell leukemia virus type 1. *J. Leukocyte Biol.* 82 44–56. 10.1189/jlb.1006641 17442856 PMC2712352

[B76] Jaworski E. Narayanan A. Van Duyne R. Shabbeer-Meyering S. Iordanskiy S. Saifuddin M. (2014). Human T-lymphotropic virus type 1-infected cells secrete exosomes that contain Tax protein. *J. Biol. Chem.* 289 22284–22305. 10.1074/jbc.M114.549659 24939845 PMC4139239

[B77] Jeang K.-T. (2001). Functional activities of the human T-cell leukemia virus type I Tax oncoprotein: cellular signaling through NF-κB. *Cytokine Growth Factor Rev.* 12 207–217. 10.1016/s1359-6101(00)00028-9 11325603

[B78] Jeffery K. J. Usuku K. Hall S. E. Matsumoto W. Taylor G. P. Procter J. (1999). HLA alleles determine human T-lymphotropic virus-I (HTLV-I) proviral load and the risk of HTLV-I-associated myelopathy. *Proc. Natl.Acad. Sci. U.S.A.* 96 3848–3853. 10.1073/pnas.96.7.3848 10097126 PMC22383

[B79] Jiang Y. Beller D. Frendl G. Graves D. (1992). Monocyte chemoattractant protein-1 regulates adhesion molecule expression and cytokine production in human monocytes. *J. Immunol.* 148 2423–2428. 1348518

[B80] Jin Z. Nagakubo D. Shirakawa A. K. Nakayama T. Shigeta A. Hieshima K. (2009). CXCR7 is inducible by HTLV-1 Tax and promotes growth and survival of HTLV-1-infected T cells. *Int. J. Cancer* 125 2229–2235. 10.1002/ijc.24612 19623653

[B81] Jones B. A. Beamer M. Ahmed S. (2010). Fractalkine/CX3CL1: a potential new target for inflammatory diseases. *Mol. Interv.* 10 263–270. 10.1124/mi.10.5.3 21045240 PMC3002219

[B82] Kagdi H. H. Demontis M. A. Fields P. A. Ramos J. C. Bangham C. R. Taylor G. P. (2017). Risk stratification of adult T-cell leukemia/lymphoma using immunophenotyping. *Cancer Med.* 6 298–309. 10.1002/cam4.928 28035765 PMC5269699

[B83] Kannagi M. Matsushita S. Harada S. (1993). Expression of the target antigen for cytotoxic T lymphocytes on adult T-cell-leukemia cells. *Int. J. Cancer* 54 582–588. 10.1002/ijc.2910540411 8514449

[B84] Kataoka K. Nagata Y. Kitanaka A. Shiraishi Y. Shimamura T. Yasunaga J. (2015). Integrated molecular analysis of adult T cell leukemia/lymphoma. *Nat. Genet.* 47 1304–1315. 10.1038/ng.3415 26437031

[B85] Katsuya H. Ishitsuka K. Utsunomiya A. Hanada S. Eto T. Moriuchi Y. (2015). Treatment and survival among 1594 patients with ATL. *Blood* 126 2570–2577. 10.1182/blood-2015-03-632489 26361794

[B86] Kawaguchi A. Orba Y. Kimura T. Iha H. Ogata M. Tsuji T. (2009). Inhibition of the SDF-1α–CXCR4 axis by the CXCR4 antagonist AMD3100 suppresses the migration of cultured cells from ATL patients and murine lymphoblastoid cells from HTLV-I Tax transgenic mice. *Blood* 114 2961–2968. 10.1182/blood-2008-11-189308 19657116

[B87] Kimura I. Tsubota T. Tada S. Sogawa J. (1986). Presence of antibodies against adult T cell leukemia antigen in the patients with chronic respiratory diseases. *Acta Med. Okayama* 40 281–284. 10.18926/amo/31916 3493619

[B88] Kira J. Nakamura M. Sawada T. Koyanagi Y. Ohori N. Itoyama Y. (1992). Antibody titers to HTLV-I-p40tax protein and gag-env hybrid protein in HTLV-I-associated myelopathy/tropical spastic paraparesis: correlation with increased HTLV-I proviral DNA load. *J. Neurol. Sci.* 107 98–104. 10.1016/0022-510x(92)90215-7 1578240

[B89] Koper O. M. Kaminska J. Sawicki K. Kemona H. (2018). CXCL9, CXCL10, CXCL11, and their receptor (CXCR3) in neuroinflammation and neurodegeneration. *Adv. Clin. Exp. Med.* 27 849–856. 10.17219/acem/68846 29893515

[B90] Kozai M. Kubo Y. Katakai T. Kondo H. Kiyonari H. Schaeuble K. (2017). Essential role of CCL21 in establishment of central self-tolerance in T cells. *J. Exp. Med.* 214 1925–1935. 10.1084/jem.20161864 28611158 PMC5502431

[B91] Kubota R. (2017). Pathogenesis of human T-lymphotropic virus type 1-associated myelopathy/tropical spastic paraparesis. *Clin. Exp. Neuroimmunol.* 8 117–128. 10.1111/cen3.12395

[B92] Kubota R. Kawanishi T. Matsubara H. Manns A. Jacobson S. (1998). Demonstration of human T lymphotropic virus type I (HTLV-I) tax-specific CD8+ lymphocytes directly in peripheral blood of HTLV-I-associated myelopathy/tropical spastic paraparesis patients by intracellular cytokine detection. *J. Immunol.* 161 482–488. 9647259

[B93] Kubota R. Umehara F. Izumo S. Ijichi S. Matsumuro K. Yashiki S. (1994). HTLV-I proviral DNA amount correlates with infiltrating CD4+ lymphocytes in the spinal cord from patients with HTLV-I-associated myelopathy. *J. Neuroimmunol.* 53 23–29. 10.1016/0165-5728(94)90060-4 7914211

[B94] Kuchroo V. K. Martin C. A. Greer J. M. Ju S. T. Sobel R. A. Dorf M. E. (1993). Cytokines and adhesion molecules contribute to the ability of myelin proteolipid protein-specific T cell clones to mediate experimental allergic encephalomyelitis. *J. Immunol.* 151 4371–4382. 7691946

[B95] LaGrenade L. Hanchard B. Fletcher V. Cranston B. Blattner W. (1990). Infective dermatitis of Jamaican children: a marker for HTLV-I infection. *Lancet* 336 1345–1347. 10.1016/0140-6736(90)92896-p 1978165

[B96] Lahrtz F. Piali L. Nadal D. Pfister H. W. Spanaus K. S. Baggiolini M. (1997). Chemotactic activity on mononuclear cells in the cerebrospinal fluid of patients with viral meningitis is mediated by interferon-γ inducible protein-10 and monocyte chemotactic protein-1. *Eur. J. Immunol.* 27 2484–2489. 10.1002/eji.1830271004 9368600

[B97] Larocca D. Chao L. A. Seto M. H. Brunck T. K. (1989). Human T-cell leukemia virus minus strand transcription in infected T-cells. *Biochem. Biophys. Res. Commun.* 163 1006–1013. 10.1016/0006-291x(89)92322-x 2476979

[B98] Lee M. Lee Y. Song J. Lee J. Chang S.-Y. (2018). Tissue-specific role of CX3CR1 expressing immune cells and their relationships with human disease. *Immune Network* 18:e5. 10.4110/in.2018.18.e5 29503738 PMC5833124

[B99] Lemasson I. Lewis M. R. Polakowski N. Hivin P. Cavanagh M. H. Thebault S. (2007). Human T-cell leukemia virus type 1 (HTLV-1) bZIP protein interacts with the cellular transcription factor CREB to inhibit HTLV-1 transcription. *J. Virol.* 81 1543–1553. 10.1128/jvi.00480-06 17151132 PMC1797566

[B100] Lepoutre V. Jain P. Quann K. Wigdahl B. Khan Z. K. (2009). Role of resident CNS cell populations in HTLV-1-associated neuroinflammatory disease. *Front. Biosci.* 14:1152–1168. 19273122 10.2741/3300 PMC2739244

[B101] Li Y. M. Pan Y. Wei Y. Cheng X. Zhou B. P. Tan M. (2004). Upregulation of CXCR4 is essential for HER2-mediated tumor metastasis. *Cancer Cell* 6 459–469. 10.1016/j.ccr.2004.09.027 15542430

[B102] Lima L. M. Cardoso L. S. Santos S. B. Oliveira R. R. Oliveira S. C. Goes A. M. (2017). Schistosoma antigens downregulate CXCL9 production by PBMC of HTLV-1-infected individuals. *Acta Trop.* 167 157–162. 10.1016/j.actatropica.2016.12.030 28040482

[B103] Liu M. T. Armstrong D. Hamilton T. A. Lane T. E. (2001). Expression of Mig (monokine induced by interferon-γ) is important in T lymphocyte recruitment and host defense following viral infection of the central nervous system. *J. Immunol.* 166 1790–1795. 10.4049/jimmunol.166.3.1790 11160225

[B104] Llano A. Esté J. (2005). Chemokines and other cytokines in human immunodeficiency virus type 1 (HIV-1) infection. *Inmunología* 24 246–260.

[B105] Loetscher M. Gerber B. Loetscher P. Jones S. A. Piali L. Clark-Lewis I. (1996). Chemokine receptor specific for IP10 and mig: structure, function, and expression in activated T-lymphocytes. *J. Exp. Med.* 184 963–969. 10.1084/jem.184.3.963 9064356 PMC2192763

[B106] Luster A. D. (1998). Chemokines—chemotactic cytokines that mediate inflammation. *N. Engl. J. Med.* 338 436–445. 10.1056/nejm199802123380706 9459648

[B107] Ma G. Yasunaga J. Fan J. Yanagawa S. Matsuoka M. (2013). HTLV-1 bZIP factor dysregulates the Wnt pathways to support proliferation and migration of adult T-cell leukemia cells. *Oncogene* 32 4222–4230. 10.1038/onc.2012.450 23045287

[B108] Ma G. Yasunaga J. Matsuoka M. (2016). Multifaceted functions and roles of HBZ in HTLV-1 pathogenesis. *Retrovirology* 13:16. 10.1186/s12977-016-0249-x 26979059 PMC4793531

[B109] Ma Q. Jones D. Borghesani P. R. Segal R. A. Nagasawa T. Kishimoto T. (1998). Impaired B-lymphopoiesis, myelopoiesis, and derailed cerebellar neuron migration in CXCR4-and SDF-1-deficient mice. *Proc. Natl. Acad. Sci. U.S.A.* 95 9448–9453. 10.1073/pnas.95.16.9448 9689100 PMC21358

[B110] Mahieux R. de Thé G. Gessain A. (1995). The tax mutation at nucleotide 7959 of human T-cell leukemia virus type 1 (HTLV-1) is not associated with tropical spastic paraparesis/HTLV-1-associated myelopathy but is linked to the cosmopolitan molecular genotype. *J. Virol.* 69 5925–5927. 10.1128/jvi.69.9.5925-5927.1995 7637041 PMC189477

[B111] Mantovani A. (1999). The chemokine system: redundancy for robust outputs. *Immunol. Today* 20 254–257. 10.1016/s0167-5699(99)01469-3 10354549

[B112] Mashino K. Sadanaga N. Yamaguchi H. Tanaka F. Ohta M. Shibuta K. (2002). Expression of chemokine receptor CCR7 is associated with lymph node metastasis of gastric carcinoma. *Cancer Res.* 62 2937–2941. 12019175

[B113] Matsuoka M. (2003). Human T-cell leukemia virus type I and adult T-cell leukemia. *Oncogene* 22:5131. 10.1038/sj.onc.1206551 12910250

[B114] Matsuoka M. Yasunaga J. (2013). Human T-cell leukemia virus type 1: replication, proliferation and propagation by Tax and HTLV-1 bZIP factor. *Curr. Opin. Virol.* 3 684–691. 10.1016/j.coviro.2013.08.010 24060211

[B115] Matsuyama W. Kawabata M. Mizoguchi A. Iwami F. Wakimoto J. Osame M. (2003). Influence of human T lymphotrophic virus type I on cryptogenic fibrosing alveolitis — HTLV-I associated fibrosing alveolitis: proposal of a new clinical entity. *Clin. Expe. Immunol.* 133 397–403. 10.1046/j.1365-2249.2003.02240.x 12930367 PMC1808791

[B116] McGrath K. E. Koniski A. D. Maltby K. M. McGann J. K. Palis J. (1999). Embryonic expression and function of the chemokine SDF-1 and its receptor. CXCR4. *Dev. Biol.* 213 442–456. 10.1006/dbio.1999.9405 10479460

[B117] Melchjorsen J. Sørensen L. N. Paludan S. R. (2003). Expression and function of chemokines during viral infections: from molecular mechanisms to in vivo function. *J. Leukocyte Biol.* 74 331–343. 10.1189/jlb.1102577 12949236 PMC7166880

[B118] Miller M. C. Mayo K. H. (2017). Chemokines from a Structural Perspective. *Int. J. Mol. Sci.* 18:E2088. 10.3390/ijms18102088 28974038 PMC5666770

[B119] Miller M. D. Krangel M. S. (1992a). Biology and biochemistry of the chemokines: a family of chemotactic and inflammatory cytokines. *Crit. Rev. Immunol.* 12 17–46. 1418604

[B120] Miller M. D. Krangel M. S. (1992b). The human cytokine I-309 is a monocyte chemoattractant. *Proc. Natl. Acad. Sci. U.S.A.* 89 2950–2954. 10.1073/pnas.89.7.2950 1557400 PMC48781

[B121] Miyagishi R. Kikuchi S. Fukazawa T. Tashiro K. (1995). Macrophage inflammatory protein-1α in the cerebrospinal fluid of patients with multiple sclerosis and other inflammatory neurological diseases. *J. Neurol. Sci.* 129 223–227. 10.1016/0022-510x(95)00004-l 7608739

[B122] Mochizuki M. Watanabe T. Yamaguchi K. Takatsuki K. Yoshimura K. Shirao M. (1992). HTLV-I uveitis: a distinct clinical entity caused by HTLV-I. *Jpn. J .Cancer Res.* 83 236–239. 10.1111/j.1349-7006.1992.tb00092.x 1582883 PMC5918816

[B123] Morgan O. S. Rodgers-Johnson P. Mora C. Char G. (1989). HTLV-1 and polymyositis in Jamaica. *Lancet* 2 1184–1187. 10.1016/s0140-6736(89)91793-5 2572904

[B124] Mori N. Gill P. S. Mougdil T. Murakami S. Eto S. Prager D. (1996). Interleukin-10 gene expression in adult T-cell leukemia. *Blood* 88 1035–1045. 10.1182/blood.v88.3.1035.bloodjournal8831035 8704212

[B125] Mori N. Krensky A. M. Ohshima K. Tomita M. Matsuda T. Ohta T. (2004). Elevated expression of CCL5/RANTES in adult T-cell leukemia cells: possible transactivation of the CCL5 gene by human T-cell leukemia virus type I tax. *Int. J. Cancer* 111 548–557. 10.1002/ijc.20266 15239133

[B126] Mori N. Mukaida N. Ballard D. W. Matsushima K. Yamamoto N. (1998). Human T-cell leukemia virus type I Tax transactivates human interleukin 8 gene through acting concurrently on AP-1 and nuclear factor-kappaB-like sites. *Cancer Res.* 58 3993–4000. 9731513

[B127] Mori N. Murakami S. Oda S. Prager D. Eto S. (1995). Production of interleukin 8 in adult T-cell leukemia cells: possible transactivation of the interleukin 8 gene by human T-cell leukemia virus type I tax. *Cancer Res.* 55 3592–3597. 7627968

[B128] Mori N. Ueda A. Ikeda S. Yamasaki Y. Yamada Y. Tomonaga M. (2000). Human T-cell leukemia virus type I tax activates transcription of the human monocyte chemoattractant protein-1 gene through two nuclear factor-kappaB sites. *Cancer Res.* 60 4939–4945. 10987310

[B129] Moriuchi H. Moriuchi M. Fauci A. S. (1998). Factors secreted by human T lymphotropic virus type I (HTLV-I)–infected cells can enhance or inhibit replication of HIV-1 in HTLV-I–uninfected cells: implications for in vivo coinfection with HTLV-I and HIV-1. *J. Exp. Med.* 187 1689–1697. 10.1084/jem.187.10.1689 9584147 PMC2212300

[B130] Moser B. Loetscher P. (2001). Lymphocyte traffic control by chemokines. *Nat. Immunol.* 2 123–128. 10.1038/84219 11175804

[B131] Mühleisen A. Giaisi M. Köhler R. Krammer P. H. Li-Weber M. (2014). Tax contributes apoptosis resistance to HTLV-1-infected T cells via suppression of Bid and Bim expression. *Cell Death Dis.* 5:e1575. 10.1038/cddis.2014.536 25522269 PMC4649845

[B132] Müller M. Carter S. Hofer M. Campbell I. (2010). Review: the chemokine receptor CXCR3 and its ligands CXCL9, CXCL10 and CXCL11 in neuroimmunity – a tale of conflict and conundrum. *Neuropathol. Appl. Neurobiol.* 36 368–387. 10.1111/j.1365-2990.2010.01089.x 20487305

[B133] Nagai M. Usuku K. Matsumoto W. Kodama D. Takenouchi N. Moritoyo T. (1998). Analysis of HTLV-I proviral load in 202 HAM/TSP patients and 243 asymptomatic HTLV-I carriers: high proviral load strongly predisposes to HAM/TSP. *J. Neurovirol.* 4 586–593. 10.3109/13550289809114225 10065900

[B134] Nagakubo D. Jin Z. Hieshima K. Nakayama T. Shirakawa A. K. Tanaka Y. (2007). Expression of CCR9 in HTLV-1+ T cells and ATL cells expressing Tax. *Int. J. Cancer* 120 1591–1597. 10.1002/ijc.22483 17205512

[B135] Nagashima K. Yoshida M. Seiki M. (1986). A single species of pX mRNA of human T-cell leukemia virus type I encodes trans-activator p40x and two other phosphoproteins. *J. Virol.* 60 394–399. 10.1128/jvi.60.2.394-399.1986 3021974 PMC288905

[B136] Naito T. Yasunaga J. I. Mitobe Y. Shirai K. Sejima H. Ushirogawa H. (2018). Distinct gene expression signatures induced by viral transactivators of different HTLV-1 subgroups that confer a different risk of HAM/TSP. *Retrovirology* 15:72. 10.1186/s12977-018-0454-x 30400920 PMC6219256

[B137] Nakagawa M. Schmitz R. Xiao W. Goldman C. K. Xu W. Yang Y. (2014). Gain-of-function CCR4 mutations in adult T cell leukemia/lymphoma. *J. Exp. Med.* 211 2497–2505. 10.1084/jem.20140987 25488980 PMC4267233

[B138] Nakayama Y. Yamazato Y. Tamayose M. Atsumi E. Yara S. Higa F. (2013). Increased expression of HBZ and Foxp3 mRNA in bronchoalveolar lavage cells taken from human T-lymphotropic virus type 1-associated lung disorder patients. *Intern. Med.* 52 2599–2609. 10.2169/internalmedicine.52.0845 24292748

[B139] Narikawa K. Fujihara K. Misu T. Feng J. Fujimori J. Nakashima I. (2005). CSF-chemokines in HTLV-I-associated myelopathy: CXCL10 up-regulation and therapeutic effect of interferon-alpha. *J. Neuroimmunol.* 159 177–182. 10.1016/j.jneuroim.2004.10.011 15652417

[B140] Nishioka K. Maruyama I. Sato K. Kitajima I. Nakajima Y. Osame M. (1989). Chronic inflammatory arthropathy associated with HTLV-I. *Lancet* 1:441 10.1016/s0140-6736(89)90038-x 2563817

[B141] Ohshima K. Kikuchi M. Masuda Y. Sumiyoshi Y. Eguchi F. Mohtai H. (1992). Human T-cell leukemia virus type I associated lymphadenitis. *Cancer* 69 239–248. 1727669 10.1002/1097-0142(19920101)69:1<239::aid-cncr2820690139>3.0.co;2-#

[B142] Osame M. Usuku K. Izumo S. Ijichi N. Amitani H. Igata A. (1986). HTLV-I associated myelopathy, a new clinical entity. *LancetLondon* 1 1031–1032. 10.1016/s0140-6736(86)91298-5 2871307

[B143] Owais M. Arya S. K. (1999). Antiviral chemokines: intracellular life of recombinant C-C chemokine RANTES. *J. Hum. Virol.* 2 270–282. 10551733

[B144] Percher F. Curis C. Pérès E. Artesi M. Rosewick N. Jeannin P. (2017). HTLV-1-induced leukotriene B4 secretion by T cells promotes T cell recruitment and virus propagation. *Nat. Commun.* 8:15890. 10.1038/ncomms15890 28639618 PMC5489682

[B145] Peterson K. E. Robertson S. J. Portis J. L. Chesebro B. (2001). Differences in cytokine and chemokine responses during neurological disease induced by polytropic murine retroviruses map to separate regions of the viral envelope gene. *J. Virol.* 75 2848–2856. 10.1128/jvi.75.6.2848-2856.2001 11222710 PMC115911

[B146] Pivarcsi A. Gombert M. Dieu-Nosjean M.-C. Lauerma A. Kubitza R. Meller S. (2004). CC chemokine ligand 18, an atopic dermatitis-associated and dendritic cell-derived chemokine, is regulated by staphylococcal products and allergen exposure. *J. Immunol.* 173 5810–5817. 10.4049/jimmunol.173.9.5810 15494534

[B147] Poiesz B. J. Ruscetti F. W. Gazdar A. F. Bunn P. A. Minna J. D. Gallo R. C. (1980). Detection and isolation of type C retrovirus particles from fresh and cultured lymphocytes of a patient with cutaneous T-cell lymphoma. *Proc. Natl.Acad. Sci. U.S.A.* 77 7415–7419. 10.1073/pnas.77.12.7415 6261256 PMC350514

[B148] Poiesz B. J. Ruscetti F. W. Reitz M. S. Kalyanaraman V. S. Gallo R. C. (1981). Isolation of a new type C retrovirus (HTLV) in primary uncultured cells of a patient with Sezary T-cell leukaemia. *Nature* 294 268–271. 10.1038/294268a0 6272125

[B149] Pozzatti R. Vogel J. Jay G. (1990). The human T-lymphotropic virus type I tax gene can cooperate with the ras oncogene to induce neoplastic transformation of cells. *Mol. Cell Biol.* 10 413–417. 10.1128/mcb.10.1.413 2403646 PMC360770

[B150] Quaresma J. A. S. Yoshikawa G. T. Koyama R. V. L. Dias G. A. S. Fujihara S. Fuzii H. T. (2016). HTLV-1, immune response and autoimmunity. *Viruses* 8:5. 10.3390/v8010005 26712781 PMC4728565

[B151] Rafatpanah H. Felegari M. Azarpazhooh M. R. Vakili R. Rajaei T. Hampson I. (2017). Altered expression of CXCR3 and CCR6 and their ligands in HTLV-1 carriers and HAM/TSP patients. *J. Med. Virol.* 89 1461–1468. 10.1002/jmv.24779 28206670

[B152] Rafatpanah H. Pravica V. FaridHosseini R. Tabatabaei A. Ollier W. Poulton K. (2007). Association between HLA-DRB1^∗^ 01 and HLA-Cw^∗^ 08 and outcome following HTLV-I infection. *Iran. J.Immunol.* 4 94–100. 17652849 10.22034/iji.2007.17185

[B153] Rajaei T. Farajifard H. Rezaee S. A. Azarpazhooh M. R. Mahmoudi M. Valizadeh N. (2018). Different roles of CXCR1 and CXCR2 in HTLV-1 carriers and HTLV-1-associated myelopathy/tropical spastic paraparesis (HAM/TSP) patients. *Med. Microbiol. Immunol.* 208 641–650. 10.1007/s00430-018-0568-8 30341468

[B154] Raman D. Sobolik-Delmaire T. Richmond A. (2011). Chemokines in health and disease. *Exp. Cell Res.* 317 575–589. 10.1016/j.yexcr.2011.01.005 21223965 PMC3063402

[B155] Romanelli L. C. F. Miranda D. M. Carneiro-Proietti A. B. F. Mamede M. Vasconcelos H. M. M. Martins M. L. (2018). Spinal cord hypometabolism associated with infection by human T-cell lymphotropic virus type 1(HTLV-1). *PLoS Negl. Trop. Dis.* 12:e0006720. 10.1371/journal.pntd.0006720 30148843 PMC6128630

[B156] Rosa D. V. Magno L. A. Pereira N. C. Romanelli L. C. Albuquerque M. R. Martins M. L. (2018). Plasma and cerebrospinal fluid levels of cytokines as disease markers of neurologic manifestation in long-term HTLV-1 infected individuals. *Biomark Med.* 12 447–454. 10.2217/bmm-2017-0313 29737866

[B157] Rösler A. Pohl M. Braune H.-J. Oertel W. Gemsa D. Sprenger H. (1998). Time course of chemokines in the cerebrospinal fluid and serum during herpes simplex type 1 encephalitis. *J Neurol. Sci.* 157 82–89. 10.1016/s0022-510x(98)00061-6 9600681

[B158] Rossi D. L. Vicari A. P. Franz-Bacon K. McClanahan T. K. Zlotnik A. (1997). Identification through bioinformatics of two new macrophage proinflammatory human chemokines: MIP-3alpha and MIP-3beta. *J. Immunol.* 158 1033–1036. 9013939

[B159] Ruckes T. Saul D. Van Snick J. Hermine O. Grassmann R. (2001). Autocrine antiapoptotic stimulation of cultured adult T-cell leukemia cells by overexpression of the chemokine I-309. *Blood* 98 1150–1159. 10.1182/blood.v98.4.1150 11493464

[B160] Sabouri A. H. Saito M. Usuku K. Bajestan S. N. Mahmoudi M. Forughipour M. (2005). Differences in viral and host genetic risk factors for development of human T-cell lymphotropic virus type 1 (HTLV-1)-associated myelopathy/tropical spastic paraparesis between Iranian and Japanese HTLV-1-infected individuals. *J. Gen. Virol.* 86 773–781. 10.1099/vir.0.80509-0 15722539

[B161] Saito M. Sejima H. Naito T. Ushirogawa H. Matsuzaki T. Matsuura E. (2017). The CC chemokine ligand (CCL) 1, upregulated by the viral transactivator Tax, can be downregulated by minocycline: possible implications for long-term treatment of HTLV-1-associated myelopathy/tropical spastic paraparesis. *Virol. J.* 14:234. 10.1186/s12985-017-0902-6 29202792 PMC5715538

[B162] Sallusto F. Lanzavecchia A. Mackay C. R. (1998a). Chemokines and chemokine receptors in T-cell priming and Th1/Th2-mediated responses. *Immunol. Today* 19 568–574. 10.1016/s0167-5699(98)01346-2 9864948

[B163] Sallusto F. Schaerli P. Loetscher P. Schaniel C. Lenig D. Mackay C. R. (1998b). Rapid and coordinated switch in chemokine receptor expression during dendritic cell maturation. *Eur. J. Immunol.* 28 2760–2769. 10.1002/(sici)1521-4141(199809)28:09¡2760::aid-immu2760¿3.0.co;2-n 9754563

[B164] Santos S. B. Oliveira P. Luna T. Souza A. Nascimento M. Siqueira I. (2012). Immunological and viral features in patients with overactive bladder associated with Human T-Cell Lymphotropic Virus Type 1 Infection. *J. Med. Virol.* 84 1809–1817. 10.1002/jmv.23341 22997085 PMC3457650

[B165] Sato T. Coler-Reilly A. Utsunomiya A. Araya N. Yagishita N. Ando H. (2013). CSF CXCL10. *PLoS Negl. Trop. Dis.* 7:e2479. 10.1371/journal.pntd.0002479 24130912 PMC3794911

[B166] Sato T. Coler-Reilly A. L. G. Yagishita N. Araya N. Inoue E. Furuta R. (2018a). Mogamulizumab (Anti-CCR4) in HTLV-1-Associated Myelopathy. *N. Engl. J. Med.* 378 529–538. 10.1056/NEJMoa1704827 29414279

[B167] Sato T. Yagishita N. Tamaki K. Inoue E. Hasegawa D. Nagasaka M. (2018b). Proposal of classification criteria for HTLV-1-associated myelopathy/tropical spastic paraparesis disease activity. *Front. Microbiol.* 9:1651. 10.3389/fmicb.2018.01651 30090093 PMC6068401

[B168] Satou Y. Utsunomiya A. Tanabe J. Nakagawa M. Nosaka K. Matsuoka M. (2012). HTLV-1 modulates the frequency and phenotype of FoxP3+CD4+ T cells in virus-infected individuals. *Retrovirology* 9:46. 10.1186/1742-4690-9-46 22647666 PMC3403885

[B169] Satou Y. Yasunaga J.-I. Yoshida M. Matsuoka M. (2006). HTLV-I basic leucine zipper factor gene mRNA supports proliferation of adult T cell leukemia cells. *Proc. Natl.Acad. Sci. U.S.A.* 103 720–725. 10.1073/pnas.0507631103 16407133 PMC1334651

[B170] Schall T. J. Bacon K. Toy K. J. Goeddel D. V. (1990). Selective attraction of monocytes and T lymphocytes of the memory phenotype by cytokine RANTES. *Nature* 347:669. 10.1038/347669a0 1699135

[B171] Schrum S. Probst P. Fleischer B. Zipfel P. F. (1996). Synthesis of the CC-chemokines MIP-1alpha, MIP-1beta, and RANTES is associated with a type 1 immune response. *J. Immunol.* 157 3598–3604. 8871660

[B172] Schutyser E. Richmond A. Van Damme J. (2005). Involvement of CC chemokine ligand 18 (CCL18) in normal and pathological processes. *J. Leukoc Biol.* 78 14–26. 10.1189/jlb.1204712 15784687 PMC2665283

[B173] Schutyser E. Struyf S. Van Damme J. (2003). The CC chemokine CCL20 and its receptor CCR6. *Cytokine Growth Factor Rev.* 14 409–426. 10.1016/s1359-6101(03)00049-2 12948524

[B174] Seki M. Kadota J. I. Higashiyama Y. Iida K. Iwashita T. Sasaki E. (1999). Elevated levels of beta-chemokines in bronchoalveolar lavage fluid (BALF) of individuals infected with human T lymphotropic virus type-1 (HTLV-1). *Clin. Exp. Immunol.* 118 417–422. 10.1046/j.1365-2249.1999.01093.x 10594561 PMC1905436

[B175] Sharma M. (2010). Chemokines and their receptors: orchestrating a fine balance between health and disease. *Crit. Rev. Biotechnol.* 30 1–22. 10.1080/07388550903187418 19780653

[B176] Sharma V. May C. C. (1999). Human T-cell lymphotrophic virus type-I tax gene induces secretion of human macrophage inflammatory protein-1α. *Biochem. Biophys. Res. Commun.* 262 429–432. 10.1006/bbrc.1999.1242 10462492

[B177] Shimauchi T. Imai S. Hino R. Tokura Y. (2005). Production of thymus and activation-regulated chemokine and macrophage-derived chemokine by CCR4+ adult T-cell leukemia cells. *Clin. Cancer Res.* 11 2427–2435. 10.1158/1078-0432.ccr-04-0491 15788694

[B178] Shimauchi T. Kabashima K. Tokura Y. (2008). Adult T-cell leukemia/lymphoma cells from blood and skin tumors express cytotoxic T lymphocyte-associated antigen-4 and Foxp3 but lack suppressor activity toward autologous CD8+ T cells. *Cancer Sci.* 99 98–106. 17970785 10.1111/j.1349-7006.2007.00646.x PMC11158631

[B179] Shimizu K. Karube K. Arakawa F. Nomura Y. Komatani H. Yamamoto K. (2007). Upregulation of CC chemokine ligand 18 and downregulation of CX3C chemokine receptor 1 expression in human T-cell leukemia virus type 1-associated lymph node lesions: Results of chemokine and chemokine receptor DNA chip analysis. *Cancer Sci.* 98 1875–1880. 10.1111/j.1349-7006.2007.00627.x 17900259 PMC11158396

[B180] Shimoyama M. Group L. S. (1991). Diagnostic criteria and classification of clinical subtypes of adult T-cell leukaemia-lymphoma: a report from the lymphoma study group (1984–87). *Br. J. Haematol.* 79 428–437. 10.1111/j.1365-2141.1991.tb08051.x 1751370

[B181] Singh S. Sadanandam A. Singh R. K. (2007). Chemokines in tumor angiogenesis and metastasis. *Cancer Metastasis Rev.* 26 453–467. 10.1007/s10555-007-9068-9 17828470 PMC4237067

[B182] Smith M. R. Greene W. C. (1991). Molecular biology of the type I human T-cell leukemia virus (HTLV-I) and adult T-cell leukemia. *J. Clin. Investig.* 87 761–766. 10.1172/jci115078 1999493 PMC329862

[B183] Starling A. L. B. Coelho-dos-Reis J. G. A. Peruhype-Magalhães V. Pascoal-Xavier M. A. Gonçalves D. U. Béla S. R. (2015). Immunological signature of the different clinical stages of the HTLV-1 infection: establishing serum biomarkers for HTLV-1-associated disease morbidity. *Biomarkers* 20 502–512. 10.3109/1354750X.2015.1094141 26474234

[B184] Strieter R. M. Polverini P. J. Kunkel S. L. Arenberg D. A. Burdick M. D. Kasper J. (1995). The functional role of the ELR motif in CXC chemokine-mediated angiogenesis. *J. Biol. Chem.* 270 27348–27357. 10.1074/jbc.270.45.27348 7592998

[B185] Sugata K. Yasunaga J.-I. Kinosada H. Mitobe Y. Furuta R. Mahgoub M. (2016). HTLV-1 Viral Factor HBZ Induces CCR4 to Promote T-cell Migration and Proliferation. *Cancer Res.* 76 5068–5079. 10.1158/0008-5472.CAN-16-0361 27402079

[B186] Tamaki K. Sato T. Tsugawa J. Fujioka S. Yagishita N. Araya N. (2019). Cerebrospinal fluid CXCL10 as a candidate surrogate marker for HTLV-1-associated myelopathy/tropical spastic paraparesis. *Front. Microbiol.* 10:2110. 10.3389/fmicb.2019.02110 31572323 PMC6749079

[B187] Tanaka A. Takahashi C. Yamaoka S. Nosaka T. Maki M. Hatanaka M. (1990). Oncogenic transformation by the tax gene of human T-cell leukemia virus type I in vitro. *Proc. Natl.Acade. Sci. U.S.A.* 87 1071–1075. 10.1073/pnas.87.3.1071 2300570 PMC53412

[B188] Tanaka Y. Mine S. Figdor C. G. Wake A. Hirano H. Tsukada J. (1998). Constitutive chemokine production results in activation of leukocyte function-associated antigen-1 on Adult T-Cell leukemia cells. *Blood* 91 3909–3919. 10.1182/blood.v91.10.3909.3909_3909_3919 9573029

[B189] Taub D. D. Conlon K. Lloyd A. R. Oppenheim J. J. Kelvin D. J. (1993). Preferential migration of activated CD4+ and CD8+ T cells in response to MIP-1 alpha and MIP-1 beta. *Science* 260 355–358. 10.1126/science.7682337 7682337

[B190] Taub D. D. Turcovski-Corrales S. M. Key M. L. Longo D. L. Murphy W. J. (1996). Chemokines and T lymphocyte activation: I. Beta chemokines costimulate human T lymphocyte activation in vitro. *J. Immunol.* 156 2095–2103. 8690897

[B191] Teruya H. Tomita M. Senba M. Ishikawa C. Tamayose M. Miyazato A. (2008). Human T-cell leukemia virus type I infects human lung epithelial cells and induces gene expression of cytokines, chemokines and cell adhesion molecules. *Retrovirology* 5:86. 10.1186/1742-4690-5-86 18808681 PMC2556696

[B192] Teuscher C. Butterfield R. J. Ma R. Z. Zachary J. F. Doerge R. W. Blankenhorn E. P. (1999). Sequence polymorphisms in the chemokines Scya1 (TCA-3), Scya2 (monocyte chemoattractant protein (MCP)-1), and Scya12 (MCP-5) are candidates for eae7, a locus controlling susceptibility to monophasic remitting/nonrelapsing experimental allergic encephalomyelitis. *J. Immunol.* 163 2262–2266. 10438970

[B193] Toulza F. Heaps A. Tanaka Y. Taylor G. P. Bangham C. R. (2008). High frequency of CD4+FoxP3+ cells in HTLV-1 infection: inverse correlation with HTLV-1-specific CTL response. *Blood* 111 5047–5053. 10.1182/blood-2007-10-118539 18094326 PMC2602587

[B194] Toulza F. Nosaka K. Takiguchi M. Pagliuca T. Mitsuya H. Tanaka Y. (2009). FoxP3+ regulatory T cells are distinct from leukemia cells in HTLV-1-associated adult T-cell leukemia. *Int. J. Cancer* 125 2375–2382. 10.1002/ijc.24664 19544530

[B195] Toulza F. Nosaka K. Tanaka Y. Schioppa T. Balkwill F. Taylor G. P. (2010). Human T-lymphotropic virus type 1-induced CC chemokine ligand 22 maintains a high frequency of functional FoxP3+ regulatory T cells. *J. Immunol.* 185 183–189. 10.4049/jimmunol.0903846 20525891 PMC3575032

[B196] Traves S. L. Smith S. J. Barnes P. J. Donnelly L. E. (2004). Specific CXC but not CC chemokines cause elevated monocyte migration in COPD: a role for CXCR2. *J. Leukocyte Biol.* 76 441–450. 10.1189/jlb.1003495 15155777

[B197] Twizere J.-C. Springael J.-Y. Boxus M. Burny A. Dequiedt F. Dewulf J.-F. (2007). Human T-cell leukemia virus type-1 Tax oncoprotein regulates G-protein signaling. *Blood* 109 1051–1060. 10.1182/blood-2006-06-026781 16990599 PMC1785145

[B198] Uchiyama T. Yodoi J. Sagawa K. Takatsuki K. Uchino H. (1977). Adult T-cell leukemia: clinical and hematologic features of 16 cases. *Blood* 50 481–492. 10.1182/blood.v50.3.481.bloodjournal503481 301762

[B199] Umehara F. Izumo S. Ronquillo A. T. Matsumuro K. Sato E. Osame M. (1994). Cytokine expression in the spinal cord lesions in HTLV-I-associated myelopathy. *J. Neuropathol. Exp. Neurol.* 53 72–77. 10.1097/00005072-199401000-00009 8301322

[B200] Umehara F. Izumo S. Takeya M. Sato E. Osame M. Takahasi K. (1996). Expression of adhesion molecules and monocyte chemoattractant protein-1 (MCP-1) in the spinal cord lesions in HTLV-I-associated myelopathy. *Acta Neuropathol.* 91 343–350. 10.1007/s004010050435 8928610

[B201] Umehara F. Okada Y. Fujimoto N. Abe M. Izumo S. Osame M. (1998). Expression of matrix metalloproteinases and tissue inhibitors of metalloproteinas HTLV-I-associated myelopathy. *J. Neuropathol. Expe. Neurol.* 57 839–849. 10.1097/00005072-199809000-00005 9737547

[B202] Utsunomiya A. Hanada S. Terada A. Kodama M. Uematsu T. Tsukasa S. (1988). Adult T-cell leukemia with leukemia cell infiltration into the gastrointestinal tract. *Cancer* 61 824–828. 10.1002/1097-0142(19880215)61:4¡824::aid-cncr2820610430¿3.0.co;2-6 3257406

[B203] Vazirinejad R. Ahmadi Z. Kazemi Arababadi M. Hassanshahi G. Kennedy D. (2014). The biological functions, structure and sources of CXCL10 and its outstanding part in the pathophysiology of multiple sclerosis. *Neuroimmunomodulation* 21 322–330. 10.1159/000357780 24642726

[B204] Vose J. Armitage J. Weisenburger D. (2008). International peripheral T-cell and natural killer/T-cell lymphoma study: pathology findings and clinical outcomes. *J. Clin. Oncol.* 26 4124–4130. 10.1200/jco.2008.16.4558 18626005

[B205] Wang J. Shiozawa Y. Wang J. Wang Y. Jung Y. Pienta K. J. (2008). The role of CXCR7/RDC1 as a chemokine receptor for CXCL12/SDF-1 in prostate cancer. *J. Biol. Chem.* 283 4283–4294. 10.1074/jbc.m707465200 18057003

[B206] Wang Z. Shang H. Jiang Y. (2017). Chemokines and chemokine receptors: accomplices for human immunodeficiency virus infection and latency. *Front. Immunol.* 8:1274. 10.3389/fimmu.2017.01274 29085362 PMC5650658

[B207] Wucherpfennig K. W. Hollsberg P. Richardson J. H. Benjamin D. Hafler D. A. (1992). T-cell activation by autologous human T-cell leukemia virus type I-infected T-cell clones. *Proc. Natl. Acad. Sci. U.S.A.* 89 2110–2114. 10.1073/pnas.89.6.2110 1549569 PMC48606

[B208] Yamaguchi K. Mochizuki M. Watanabe T. Yoshimura K. Shirao M. Araki S. (1994). Human T lymphotropic virus type 1 uveitis after Graves disease. *Br. J. Ophthalmol.* 78 163–166. 10.1136/bjo.78.3.163 8148330 PMC504729

[B209] Yamamoto K. Utsunomiya A. Tobinai K. Tsukasaki K. Uike N. Uozumi K. (2010). Phase I study of KW-0761, a defucosylated humanized anti-CCR4 antibody, in relapsed patients with adult T-cell leukemia-lymphoma and peripheral T-cell lymphoma. *J. Clin. Oncol.* 28 1591–1598. 10.1200/JCO.2009.25.3575 20177026

[B210] Yamamoto M. Matsuyama W. Oonakahara K. Watanabe M. Higashimoto I. Kawabata M. (2004). Influence of human T lymphotrophic virus type I on diffuse pan-bronchiolitis. *Clin. Exp. Immunol*, 136 513–520. 10.1111/j.1365-2249.2004.02485.x 15147354 PMC1809062

[B211] Yamamoto-Taguchi N. Satou Y. Miyazato P. Ohshima K. Nakagawa M. Katagiri K. (2013). HTLV-1 bZIP factor induces inflammation through labile Foxp3 expression. *PLoS pathog.* 9:e1003630. 10.1371/journal.ppat.1003630 24068936 PMC3777874

[B212] Yamano Y. Araya N. Sato T. Utsunomiya A. Azakami K. Hasegawa D. (2009). Abnormally High Levels of Virus-Infected IFN-γ(+)CCR4(+)CD4(+)CD25(+) T cells in a retrovirus-associated neuroinflammatory disorder. *PLoS One* 4:e6517. 10.1371/journal.pone.0006517 19654865 PMC2715877

[B213] Yamano Y. Coler-Reilly A. (2017). HTLV-1 induces a Th1-like state in CD4(+)CCR4(+) T cells that produces an inflammatory positive feedback loop via astrocytes in HAM/TSP. *J. Neuroimmunol.* 304 51–55. 10.1016/j.jneuroim.2016.08.012 27542993

[B214] Yamazato Y. Miyazato A. Kawakami K. Yara S. Kaneshima H. Saito A. (2003). High expression of p40(tax) and pro-inflammatory cytokines and chemokines in the lungs of human T-lymphotropic virus type 1-related bronchopulmonary disorders. *Chest* 124 2283–2292. 10.1378/chest.124.6.2283 14665512

[B215] Yang J. Richmond A. (2004). The angiostatic activity of interferon-inducible protein-10/CXCL10 in human melanoma depends on binding to CXCR3 but not to glycosaminoglycan. *Mol. Ther. J. Am. Soc. Gene Ther.* 9 846–855. 10.1016/j.ymthe.2004.01.010 15194051 PMC2668261

[B216] Yoshida M. (2001). Multiple viral strategies of HTLV-1 for dysregulation of cell growth control. *Annu. Rev. Immunol.* 19 475–496. 10.1146/annurev.immunol.19.1.475 11244044

[B217] Yoshida M. Miyoshi I. Hinuma Y. (1982). Isolation and characterization of retrovirus from cell lines of human adult T-cell leukemia and its implication in the disease. *Proc. Natl. Acad. Sci. U.S.A.* 79 2031–2035. 10.1073/pnas.79.6.2031 6979048 PMC346116

[B218] Yoshie O. Fujisawa R. Nakayama T. Harasawa H. Tago H. Izawa D. (2002). Frequent expression of CCR4 in adult T-cell leukemia and human T-cell leukemia virus type 1–transformed T cells. *Blood* 99 1505–1511. 10.1182/blood.v99.5.1505 11861261

[B219] Yoshie O. Imai T. Nomiyama H. (2001). Chemokines in immunity. *Adv. Immunol.* 78 57–110. 11432208 10.1016/s0065-2776(01)78002-9

[B220] Youn B.-S. Kim Y. J. Mantel C. Yu K.-Y. Broxmeyer H. E. (2001). Blocking of c-FLIPL–independent cycloheximide-induced apoptosis or Fas-mediated apoptosis by the CC chemokine receptor 9/TECK interaction. *Blood* 98 925–933. 10.1182/blood.v98.4.925 11493434

[B221] Zhao T. (2016). The Role of HBZ in HTLV-1-Induced Oncogenesis. *Viruses* 8:E34. 10.3390/v8020034 26848677 PMC4776189

[B222] Zhao T. Yasunaga J. Satou Y. Nakao M. Takahashi M. Fujii M. (2009). Human T-cell leukemia virus type 1 bZIP factor selectively suppresses the classical pathway of NF-kappaB. *Blood* 113 2755–2764. 10.1182/blood-2008-06-161729 19064727

[B223] Zlotnik A. Yoshie O. (2000). Chemokines: a new classification system and their role in immunity. *Immunity* 12 121–127. 10.1016/s1074-7613(00)80165-x 10714678

