# Supplementary material for: The Role of Chemokines in the Pathogenesis of HTLV-1
Source: Front Microbiol. 2020 Mar 13;11:421. doi: 10.3389/fmicb.2020.00421 (PMC7083101; doi:10.3389/fmicb.2020.00421)
Supplement: Supplementary file 1 [file Table_1.DOCX]

Supplementary Material

**Supplementary Table**

Table 1. The role of chemokines and chemokine receptors (Chemokine/receptor axes) in HTLV-1 pathogenesis.

| Chemokine/ Receptor | HAM/TSP patients | ATL patients | HTLV-1-infectedT Cell lines | Function | Ref |
| --- | --- | --- | --- | --- | --- |
| CCL1 | ↑ | ↑ |  | -Apoptosis inhibition  -Leukocyte attraction  -CNS inflammation | (Ruckes et al., 2001; Saito et al., 2017) |
| CCL2 | ↓ | ↑ |  | Type 2 Immune response | (Mori et al., 2000; Narikawa et al., 2005; Guerreiro et al., 2006) |
| CCL3 | ↑ | ↑ |  | -Leukocyte attraction  -Adhesion and tissue infiltration | (Taub et al., 1993; BERTINI et al., 1995; Biddison et al., 1997; Tanaka et al., 1998) |
| CCL4 | ↑ | ↑ |  | -Leukocyte attraction  -Adhesion and tissue infiltration | (Taub et al., 1993; BERTINI et al., 1995; Biddison et al., 1997; Tanaka et al., 1998) |
| CCL5 | ↑ | ↑ | ↑ | -Leukocyte attraction  -Type 1 immune response  -Tissue infiltration | (Schrum et al., 1996; Mori et al., 2004; Tanaka et al., 2008; Amorim et al., 2014) |
| CCL17 |  | ↑ |  | Skin infiltration | (Shimauchi et al., 2005) |
| CCL18 |  | ↑ |  | -Attraction and activation of T cells  -Initiation of Immune response | (Shimizu et al., 2007; Ruytinx et al., 2018) |
| CCL19 |  | ↑ |  | Leukocyte recruitment and homing | (Kozai et al., 2017) |
| CCL20 | ↑ |  | ↑ | -DC/lymphocyte recruitment  -Inflammation | (Imaizumi et al., 2002; Teruya et al., 2008) |
| CCL21 |  | ↑ |  | -Leukocyte recruitment and homing | (Kozai et al., 2017) |
| CCL22 |  | ↑ | ↑ | -Transmission of virus to CCR4^+^ CD4^+^ T cells | (Shimauchi et al., 2005; Hieshima et al., 2008; Toulza et al., 2010) |
| CCL24 | ↓ |  |  | Type 2 immune response | (Guerreiro et al., 2006) |
| CCL25 |  | ↑ |  | -Leukocyte infiltration into GIT  -Apoptosis inhibition | (Youn et al., 2001; Nagakubo et al., 2007) |
| CCR4 | ↑ | ↑ | ↑ | -Th2 immune response  -Tissue infiltration  -CNS inflammation | (Yoshie et al., 2002; Shimauchi et al., 2005; Yamano et al., 2009; Araya et al., 2014; Yamano and Coler-Reilly, 2017) |
| CCR5 |  | ↑ |  | Tissue infiltration | (Mori et al., 2004; Sadaghiani et al., 2019) |
| CCR6 |  |  | ↑ | Leukocyte recruitment | (Baba et al., 1997; Rafatpanah et al., 2017) |
| CCR7 |  | ↑ |  | Lymphoid tissue infiltration of Leukocytes | (Hasegawa et al., 2000; Kozai et al., 2017) |
| CCR8 | ↑ | ↑ | ↑ | -Monocyte recruitment  -Apoptosis inhibition | (Ruckes et al., 2001; Saito et al., 2017) |
| CCR9 |  | ↑ | ↑ | Gastrointestinal infiltration | (Nagakubo et al., 2007) |
| CXCL8 | ↑ | ↑ |  | Leukocyte attraction | (Mori et al., 1995; Chaves et al., 2016) |
| CXCL9 | ↑ |  |  | -Leukocyte recruitment  -CNS inflammation | (Sato et al., 2013; Chaves et al., 2016; Futsch et al., 2018; Guerra et al., 2018) |
| CXCL10 | ↑ |  |  | -Leukocyte recruitment  -CNS inflammation | (Sato et al., 2013; Chaves et al., 2016; Futsch et al., 2018; Guerra et al., 2018; Tamaki et al., 2019) |
| CXCL12 |  | ↑ | ↑ | -Leukocyte migration  -Tissue infiltration | (Kawaguchi et al., 2009) |
| CXCR4 |  | ↑ |  | -Leukocyte migration  -Tissue infiltration | (Kawaguchi et al., 2009) |
| CXCR7 |  | ↑ | ↑ | Cell growth and survival | (Jin et al., 2009) |
| CX3CL1/ CX3CR1 |  | ↓ |  | Leukocyte attraction and adhesion | (Shimizu et al., 2007) |

Abbreviations: ATL, Adult T-cell leukemia/lymphoma; CCL, Chemokine ligand C; CCR, Chemokine receptor C; CNS, Central nervous system; CX3CL, Chemokine ligand CX3C; CX3CR, Chemokine receptor CX3C; CXCL, Chemokine ligand CXC; CXCR, Chemokine receptor CXC; DC, Dendritic cell; GIT, Gastrointestinal tract; HAM/TSP, HTLV-associated myelopathy/tropical spastic paraparesis; HTLV-1, Human T cell leukemia virus type 1; Th2, Type 2 T helper cells.

Amorim, C.F., Souza, A.S., Diniz, A.G., Carvalho, N.B., Santos, S.B., and Carvalho, E.M. (2014). Functional activity of monocytes and macrophages in HTLV-1 infected subjects. *PLoS Negl Trop Dis* 8(12)**,** e3399. doi: 10.1371/journal.pntd.0003399.

Araya, N., Sato, T., Ando, H., Tomaru, U., Yoshida, M., Coler-Reilly, A., et al. (2014). HTLV-1 induces a Th1-like state in CD4+CCR4+ T cells. *J Clin Invest* 124(8)**,** 3431-3442. doi: 10.1172/jci75250.

Baba, M., Imai, T., Nishimura, M., Kakizaki, M., Takagi, S., Hieshima, K., et al. (1997). Identification of CCR6, the specific receptor for a novel lymphocyte-directed CC chemokine LARC. *J Biol Chem* 272(23)**,** 14893-14898.

BERTINI, R., LUINI, W., SOZZANI, S., BOTTAZZI, B., RUGGIERO, P., BORASCHI, D., et al. (1995). Identification of MIP-1α/LD78 as a monocyte chemoattractant released by the HTLV-I-transformed cell line MT4. *AIDS research and human retroviruses* 11(1)**,** 155-160.

Biddison, W.E., Kubota, R., Kawanishi, T., Taub, D.D., Cruikshank, W.W., Center, D.M., et al. (1997). Human T cell leukemia virus type I (HTLV-I)-specific CD8+ CTL clones from patients with HTLV-I-associated neurologic disease secrete proinflammatory cytokines, chemokines, and matrix metalloproteinase. *J Immunol* 159(4)**,** 2018-2025.

Chaves, D.G., Sales, C.C., de Cassia Goncalves, P., da Silva-Malta, M.C., Romanelli, L.C., Ribas, J.G., et al. (2016). Plasmatic proinflammatory chemokines levels are tricky markers to monitoring HTLV-1 carriers. *J Med Virol* 88(8)**,** 1438-1447. doi: 10.1002/jmv.24481.

Futsch, N., Prates, G., Mahieux, R., Casseb, J., and Dutartre, H. (2018). Cytokine Networks Dysregulation during HTLV-1 Infection and Associated Diseases. *Viruses* 10(12). doi: 10.3390/v10120691.

Guerra, M., Luna, T., Souza, A., Amorim, C., Carvalho, N.B., Carvalho, L., et al. (2018). Local and systemic production of proinflammatory chemokines in the pathogenesis of HAM/TSP. *Cell Immunol* 334**,** 70-77. doi: 10.1016/j.cellimm.2018.09.009.

Guerreiro, J., Santos, S., Morgan, D., Porto, A., Muniz, A., Ho, J., et al. (2006). Levels of serum chemokines discriminate clinical myelopathy associated with human T lymphotropic virus type 1 (HTLV‐1)/tropical spastic paraparesis (HAM/TSP) disease from HTLV‐1 carrier state. *Clinical & Experimental Immunology* 145(2)**,** 296-301.

Hasegawa, H., Nomura, T., Kohno, M., Tateishi, N., Suzuki, Y., Maeda, N., et al. (2000). *Hasegawa H, Nomura T, Kohno M, Tateishi N, Suzuki Y, Maeda N, Fujisawa R, Yoshie O, Fujita SIncreased chemokine receptor CCR7/EBI1 expression enhances the infiltration of lymphoid organs by adult T-cell leukaemia cells. Blood 95: 30-38.*

Hieshima, K., Nagakubo, D., Nakayama, T., Shirakawa, A.-K., Jin, Z., and Yoshie, O. (2008). Tax-inducible production of CC chemokine ligand 22 by human T cell leukemia virus type 1 (HTLV-1)-infected T cells promotes preferential transmission of HTLV-1 to CCR4-expressing CD4+ T cells. *The Journal of Immunology* 180(2)**,** 931-939.

Imaizumi, Y., Sugita, S., Yamamoto, K., Imanishi, D., Kohno, T., Tomonaga, M., et al. (2002). Human T cell leukemia virus type-I Tax activates human macrophage inflammatory protein-3α/CCL20 gene transcription via the NF-κB pathway. *International immunology* 14(2)**,** 147-155.

Jin, Z., Nagakubo, D., Shirakawa, A.K., Nakayama, T., Shigeta, A., Hieshima, K., et al. (2009). CXCR7 is inducible by HTLV‐1 Tax and promotes growth and survival of HTLV‐1‐infected T cells. *International Journal of Cancer* 125(9)**,** 2229-2235.

Kawaguchi, A., Orba, Y., Kimura, T., Iha, H., Ogata, M., Tsuji, T., et al. (2009). Inhibition of the SDF-1α–CXCR4 axis by the CXCR4 antagonist AMD3100 suppresses the migration of cultured cells from ATL patients and murine lymphoblastoid cells from HTLV-I Tax transgenic mice. *Blood* 114(14)**,** 2961-2968.

Kozai, M., Kubo, Y., Katakai, T., Kondo, H., Kiyonari, H., Schaeuble, K., et al. (2017). Essential role of CCL21 in establishment of central self-tolerance in T cells. *The Journal of Experimental Medicine* 214(7)**,** 1925-1935. doi: 10.1084/jem.20161864.

Mori, N., Krensky, A.M., Ohshima, K., Tomita, M., Matsuda, T., Ohta, T., et al. (2004). Elevated expression of CCL5/RANTES in adult T-cell leukemia cells: possible transactivation of the CCL5 gene by human T-cell leukemia virus type I tax. *Int J Cancer* 111(4)**,** 548-557. doi: 10.1002/ijc.20266.

Mori, N., Murakami, S., Oda, S., Prager, D., and Eto, S. (1995). Production of interleukin 8 in adult T-cell leukemia cells: possible transactivation of the interleukin 8 gene by human T-cell leukemia virus type I tax. *Cancer Res* 55(16)**,** 3592-3597.

Mori, N., Ueda, A., Ikeda, S., Yamasaki, Y., Yamada, Y., Tomonaga, M., et al. (2000). Human T-cell leukemia virus type I tax activates transcription of the human monocyte chemoattractant protein-1 gene through two nuclear factor-kappaB sites. *Cancer Res* 60(17)**,** 4939-4945.

Nagakubo, D., Jin, Z., Hieshima, K., Nakayama, T., Shirakawa, A.K., Tanaka, Y., et al. (2007). Expression of CCR9 in HTLV‐1+ T cells and ATL cells expressing Tax. *International journal of cancer* 120(7)**,** 1591-1597.

Narikawa, K., Fujihara, K., Misu, T., Feng, J., Fujimori, J., Nakashima, I., et al. (2005). CSF-chemokines in HTLV-I-associated myelopathy: CXCL10 up-regulation and therapeutic effect of interferon-alpha. *J Neuroimmunol* 159(1-2)**,** 177-182. doi: 10.1016/j.jneuroim.2004.10.011.

Rafatpanah, H., Felegari, M., Azarpazhooh, M.R., Vakili, R., Rajaei, T., Hampson, I., et al. (2017). Altered expression of CXCR3 and CCR6 and their ligands in HTLV-1 carriers and HAM/TSP patients. *J Med Virol* 89(8)**,** 1461-1468. doi: 10.1002/jmv.24779.

Ruckes, T., Saul, D., Van Snick, J., Hermine, O., and Grassmann, R. (2001). Autocrine antiapoptotic stimulation of cultured adult T-cell leukemia cells by overexpression of the chemokine I-309. *Blood* 98(4)**,** 1150-1159.

Ruytinx, P., Proost, P., Van Damme, J., and Struyf, S. (2018). Chemokine-Induced Macrophage Polarization in Inflammatory Conditions. *Front Immunol* 9**,** 1930. doi: 10.3389/fimmu.2018.01930.

Sadaghiani, N.H., Pirayeshfard, L., Aghaie, A., and Sharifi, Z. (2019). The Effect of TAX-1 Gene of Human T-cell Leukemia Virus Type-1 on the Expression of CCR5 in K562 Cell Line. *Avicenna journal of medical biotechnology* 11(1)**,** 67.

Saito, M., Sejima, H., Naito, T., Ushirogawa, H., Matsuzaki, T., Matsuura, E., et al. (2017). The CC chemokine ligand (CCL) 1, upregulated by the viral transactivator Tax, can be downregulated by minocycline: possible implications for long-term treatment of HTLV-1-associated myelopathy/tropical spastic paraparesis. *Virology Journal* 14(1)**,** 234. doi: 10.1186/s12985-017-0902-6.

Sato, T., Coler-Reilly, A., Utsunomiya, A., Araya, N., Yagishita, N., Ando, H., et al. (2013). CSF CXCL10, CXCL9, and Neopterin as Candidate Prognostic Biomarkers for HTLV-1-Associated Myelopathy/Tropical Spastic Paraparesis. *PLoS Neglected Tropical Diseases* 7(10)**,** e2479. doi: 10.1371/journal.pntd.0002479.

Schrum, S., Probst, P., Fleischer, B., and Zipfel, P.F. (1996). Synthesis of the CC-chemokines MIP-1alpha, MIP-1beta, and RANTES is associated with a type 1 immune response. *J Immunol* 157(8)**,** 3598-3604.

Shimauchi, T., Imai, S., Hino, R., and Tokura, Y. (2005). Production of thymus and activation-regulated chemokine and macrophage-derived chemokine by CCR4+ adult T-cell leukemia cells. *Clinical cancer research* 11(6)**,** 2427-2435.

Shimizu, K., Karube, K., Arakawa, F., Nomura, Y., Komatani, H., Yamamoto, K., et al. (2007). Upregulation of CC chemokine ligand 18 and downregulation of CX3C chemokine receptor 1 expression in human T‐cell leukemia virus type 1‐associated lymph node lesions: Results of chemokine and chemokine receptor DNA chip analysis. *Cancer science* 98(12)**,** 1875-1880.

Tamaki, K., Sato, T., Tsugawa, J., Fujioka, S., Yagishita, N., Araya, N., et al. (2019). Cerebrospinal Fluid CXCL10 as a Candidate Surrogate Marker for HTLV-1-Associated Myelopathy/Tropical Spastic Paraparesis. *Front Microbiol* 10**,** 2110. doi: 10.3389/fmicb.2019.02110.

Tanaka, M., Matsushita, T., Tateishi, T., Ochi, H., Kawano, Y., Mei, F.-J., et al. (2008). Distinct CSF cytokine/chemokine profiles in atopic myelitis and other causes of myelitis. *Neurology* 71(13)**,** 974-981.

Tanaka, Y., Mine, S., Figdor, C.G., Wake, A., Hirano, H., Tsukada, J., et al. (1998). Constitutive Chemokine Production Results in Activation of Leukocyte Function-Associated Antigen-1 on Adult T-Cell Leukemia Cells. *Blood* 91(10)**,** 3909-3919.

Taub, D.D., Conlon, K., Lloyd, A.R., Oppenheim, J.J., and Kelvin, D.J. (1993). Preferential migration of activated CD4+ and CD8+ T cells in response to MIP-1 alpha and MIP-1 beta. *Science* 260(5106)**,** 355-358.

Teruya, H., Tomita, M., Senba, M., Ishikawa, C., Tamayose, M., Miyazato, A., et al. (2008). Human T-cell leukemia virus type I infects human lung epithelial cells and induces gene expression of cytokines, chemokines and cell adhesion molecules. *Retrovirology* 5(1)**,** 86.

Toulza, F., Nosaka, K., Tanaka, Y., Schioppa, T., Balkwill, F., Taylor, G.P., et al. (2010). Human T-lymphotropic virus type 1-induced CC chemokine ligand 22 maintains a high frequency of functional FoxP3+ regulatory T cells. *J Immunol* 185(1)**,** 183-189. doi: 10.4049/jimmunol.0903846.

Yamano, Y., Araya, N., Sato, T., Utsunomiya, A., Azakami, K., Hasegawa, D., et al. (2009). Abnormally High Levels of Virus-Infected IFN-γ(+)CCR4(+)CD4(+)CD25(+) T Cells in a Retrovirus-Associated Neuroinflammatory Disorder. *PLoS ONE* 4(8)**,** e6517. doi: 10.1371/journal.pone.0006517.

Yamano, Y., and Coler-Reilly, A. (2017). HTLV-1 induces a Th1-like state in CD4(+)CCR4(+) T cells that produces an inflammatory positive feedback loop via astrocytes in HAM/TSP. *J Neuroimmunol* 304**,** 51-55. doi: 10.1016/j.jneuroim.2016.08.012.

Yoshie, O., Fujisawa, R., Nakayama, T., Harasawa, H., Tago, H., Izawa, D., et al. (2002). Frequent expression of CCR4 in adult T-cell leukemia and human T-cell leukemia virus type 1–transformed T cells. *Blood* 99(5)**,** 1505-1511.

Youn, B.-S., Kim, Y.J., Mantel, C., Yu, K.-Y., and Broxmeyer, H.E. (2001). Blocking of c-FLIPL–independent cycloheximide-induced apoptosis or Fas-mediated apoptosis by the CC chemokine receptor 9/TECK interaction. *Blood* 98(4)**,** 925-933.
